# Gene networks in *Drosophila melanogaster*: integrating experimental data to predict gene function

**DOI:** 10.1186/gb-2009-10-9-r97

**Published:** 2009-09-16

**Authors:** James C Costello, Mehmet M Dalkilic, Scott M Beason, Jeff R Gehlhausen, Rupali Patwardhan, Sumit Middha, Brian D Eads, Justen R Andrews

**Affiliations:** 1 School of Informatics, Indiana University, E. Tenth St, Bloomington, Indiana 47408, USA; 2 Department of Biology, Indiana University, E. Third St, Bloomington, Indiana 47405, USA; 3 Center for Genomics and Bioinformatics, Indiana University, E. Third St., Bloomington, Indiana 47405, USA; 4 Current address: Department of Genome Sciences, University of Washington, NE Pacific St, Seattle, Washington 98195-5065, USA; 5 Current address: Bioinformatics Core, Mayo Clinic, First St SW, Rochester, Minnesota 55905, USA

## Abstract

**Background:**

Discovering the functions of all genes is a central goal of contemporary biomedical research. Despite considerable effort, we are still far from achieving this goal in any metazoan organism. Collectively, the growing body of high-throughput functional genomics data provides evidence of gene function, but remains difficult to interpret.

**Results:**

We constructed the first network of functional relationships for *Drosophila melanogaster *by integrating most of the available, comprehensive sets of genetic interaction, protein-protein interaction, and microarray expression data. The complete integrated network covers 85% of the currently known genes, which we refined to a high confidence network that includes 20,000 functional relationships among 5,021 genes. An analysis of the network revealed a remarkable concordance with prior knowledge. Using the network, we were able to infer a set of high-confidence Gene Ontology biological process annotations on 483 of the roughly 5,000 previously unannotated genes. We also show that this approach is a means of inferring annotations on a class of genes that cannot be annotated based solely on sequence similarity. Lastly, we demonstrate the utility of the network through reanalyzing gene expression data to both discover clusters of coregulated genes and compile a list of candidate genes related to specific biological processes.

**Conclusions:**

Here we present the the first genome-wide functional gene network in *D. melanogaster*. The network enables the exploration, mining, and reanalysis of experimental data, as well as the interpretation of new data. The inferred annotations provide testable hypotheses of previously uncharacterized genes.

## Abstract 2

The first computational interaction network built from *Drosophila melanogaster* protein-protein and genetic interaction data allows the functional annotation of orphan genes and reveals clusters of functionally-related genes.

## Background

Understanding how a metazoan organism functions requires knowledge of the biochemical, cellular, and overall phenotypic effects of all genes. Despite considerable effort, direct experimental evidence supporting the participation of genes in biological process(es) exists for only a modest proportion of the full complement of metazoan genes (as reflected by Gene Ontology (GO) annotations [1]; see Materials and methods section for details). For instance, of the nearly 29 *K *(*K *= 1,000) genes in mouse, there is experimental evidence supporting the functional annotation of less than half, or approximately 12 *K *genes. Similarly, for *Caenorhabditis elegans*, experimental evidence exists for about a third (approximately 7.5 *K*) of its approximately 20 *K *genes. Even the most experimentally amenable and well-characterized eukaryotic organism, *Saccharomyces cerevisiae*, though not a metazoan, still has over 1 *K *of its 6 *K *genes lacking functional annotation [2].

Both new and improving synthetic and analytic genome-scale technologies can help us determine the biological process(es) of unannotated genes, as well as provide new insight into annotated genes. Some of these approaches include yeast-two-hybrid (Y2H) screens to detect physically interacting proteins, expression profiling to detect transcript coexpression, modifier screens to identify genetic interactions, RNA interference screens to measure the genetic effects of gene knockdowns, genome tiling path arrays and next-gen sequencing to discover transcribed genomic elements, and ChIP-Chip and ChIP-seq to identify protein-DNA interactions. While these assays have the advantage of being high-throughput, distinguishing the biologically relevant relationships from noise within a single experiment is not a straightforward task. This, together with their sheer volume, makes interpretation challenging.

Methods to derive functional annotation from the available corpuses of data have been developed [3,4] and those that focus on data integration are among the more successful [5-9]. Integrating different types of genomics data has been shown to reveal relationships between genes not distinguishable within single datasets [10,11]. In the context of genomics data, the overarching theme of an integrative model is to distill the available data down to a value indicative of a gene pair being functionally related. These methods, pioneered by Troyanskaya *et al*. [5], Jansen *et al*. [8], and Lee *et al*. [12], were heavily based on Bayesian networks to bring together weighted gene-gene relationships across heterogeneous datasets. Here, and inspired from this previous work, a functional relationship between genes represents the likelihood that two genes are involved in the same biological process. Integrative models have been successfully used to construct molecular networks (that is, transcriptional regulation and metabolic) [13,14], predict genetic interactions in yeast [15], predict phenotypic effects in worm [16], provide new gene candidates in human disease [17-20], and make novel predictions of gene function [6,12,21-27]. The number of organisms with well-annotated genomes and sufficient experimental data to build integrated networks is limited. Thus, networks constructed from genome-wide data have been restricted to: bacteria [14,25], *S. cerevisiae *[5,12,26,28,29], *C. elegans *[16,30], mouse [31,32], and human [18-20,27]. *Drosophila *is among the most well-annotated organisms, and the amount of experimental and computational data for it is on par with worm, yeast, and mouse [33,34]. Although there exist repositories for flies that provide sophisticated query capability, namely FlyBase [35] and FlyMine [36], as well as ongoing attempts at mining disparate sources of fly data [21,37,38], an integrated system that can be interrogated *ad hoc *to easily deal with large sets of *Drosophila *genes has not been available until now.

As one of the preeminent model organisms, *Drosophila *has been the object of study for more than a century [39]. This research has not only increased our understanding of the organism itself [40,41], but more importantly increased our knowledge of molecular mechanisms in biology in its broadest sense, particularly in the fields of genetics, development, evolution, and molecular biology. *Drosophila *has the richest set of sequenced genomes for a metazoan genus [42,43] and, along with *C. elegans *and human, will have the most comprehensive inventory of metazoan genomic elements stemming from the modENCODE [44] and ENCODE projects [45]. Despite these resources, there exist many genes for which biological process(es) are unknown. At the time of this study (v5.3 of the *D. melanogaster *genome [46]) there is direct experimental evidence supporting the biological process GO annotations (hereafter referred to as GO:BP) for less than half (approximately 42%) of the more than 15 *K *protein-coding genes (counted from curator reviewed GO evidence codes). These annotations are mostly based on genetic evidence, (that is, mutant phenotypes, genetic interactions, and RNA interference knockdown phenotypes). In addition to experimental evidence, roughly 26% of the genes have GO:BP terms that are inferred from electronic annotation methods (inferred from electronic annotation (IEA) GO evidence code). Considering all the available methods to determine in which biological process(es) a gene participates, we underscore the fact that nearly one-third of *Drosophila *protein-coding genes (> 4.6 *K*) remain unannotated.

In this study, we bring together experimental data to build the first integrated functional gene networks in *Drosophila*. We focus specifically on building functional relationships between pairs of genes that are likely to participate in the same biological process and are supported by experimental evidence. We adapt the approach developed by Marcotte and colleagues [12,16,28] to integrate three experimental classes of data, in particular, genetic interactions, protein-protein interactions, and microarray gene expression. We demonstrate that the integrated networks perform well at recapitulating known functional relationships and outperform networks built exclusively from individual types of data (that is, just microarray data). We then utilize the functional relationships in the network to predict GO:BP annotations for unannotated genes using the Markov random field (MRF) method [47] and demonstrate that this approach performs well at predicting annotations through tenfold cross-validation. We use this method to infer high confidence GO:BP terms for 483 uncharacterized genes, and evaluate these predictions with respect to the available independent evidence. Finally, we use the constructed network to reanalyze gene expression data related to nutritional deprivation. We show that the network can be used to discover clusters of functionally related genes amongst genes that were identified to be differentially expressed.

All data are made available through supplemental material [48].

## Results

### Types of data and datasets

This study includes three classes of data: genetic interactions (GIs); protein-protein interactions (PPIs); and microarray (MA) expression data. All reported GIs were downloaded from FlyBase [46] and each GI was weighted equally. PPIs were extracted from the following databases: BIND [49], DIP [50], DroID [51], BioGRID [52], and IntAct [53]. The union of the PPIs across these databases was taken and separated based on the assay type, namely direct assay (that is, co-immunoprecipitation, biochemical assay), high-confidence Y2H (high-confidence as defined by Giot *et al*. [54]), and positive Y2H. A total of 18 published MA experiments were used (see Figure S1 at [55]). These 18 experiments can be divided into individual subcomponents, often reflecting several timecourse studies done under the umbrella of one published experiment. Thus, these 18 experiments were broken into 34 individual datasets. The 34 datasets were evaluated using log-likelihood scores (*LLS*) and several other filters detailed in the 'Calculating the likelihood that gene pairs participate in a common biological process' and Materials and methods sections. From these results, we determined that 20 of the 34 datasets provided *LLS*s meeting our evaluation criteria; therefore, only these 20 MA datasets were included in the construction of the integrated networks. In total, 24 datasets were used in this study, including all GIs, three classes of PPIs, and 20 MA datasets (see Table S4 at [55] for the number of conditions per MA dataset). The datasets are summarized in Table 1, and further details of the acquisition and processing of these datasets are provided in the Materials and methods section.

**Table 1:** Datasets

**Source**	**Dataset**	**Pass filter?**	**Genes**	**Relationships**
**Genetic interactions**				
FlyBase	All reported GIs	N/A	2,878	6,941
				
**Protein-protein interactions**				
BIND, DIP, IntAct	Direct assay	N/A	935	1,234
DroID, BioGRID	High-confidence Y2H	N/A	4,543	4,590
	Positive Y2H	N/A	6,183	19,584
				
**Microarray**				
Hooper *et al*. [100]	All conditions	Yes	10,460	3,289,275
Chintapalli *et al*. [102]	All conditions	Yes	10,054	3,618,216
Parisi *et al*. [92]	All conditions	Yes	9,922	5,656,854
Edwards *et al*. [99]	Line1	Yes	8,403	8,072,394
	Line2	Yes	8,296	8,118,665
	All conditions	Yes	0	0
Altenhein *et al*. [98]	All conditions	Yes	8,341	1,030,457
	Gof	No	0	0
	Lof	No	0	0
Hild *et al*. [97]	All conditions	Yes	8,214	677,746
Qin *et al*. [94]	All conditions	Yes	6,734	4,187,496
Tomancak *et al*. [103]	All conditions	Yes	6,288	2,626,310
Magalhaes *et al*. [59]	All conditions	Yes	5,718	1,102,629
De Gregorio *et al*. [57]	All conditions	Yes	5,698	1,561,265
	Bacteria	Yes	4,920	237,361
	Fungus	No	0	0
	*Spaetzle*	No	0	0
	*Relish*	No	0	0
	*Spaetzle *&*relish*	No	0	0
Sandmann *et al*. [101]	All conditions	Yes	5,474	1,238,924
Arbeitman *et al*. [61]	All conditions	Yes	4,354	1,769,479
	Embryo	Yes	4,126	1,271,286
	Larva	No	0	0
	Pupal	No	0	0
	Adult male	No	0	0
	Adult female	No	0	0
Sorensen *et al*. [96]	Heat	Yes	4,219	690,181
	No heat	Yes	4,083	701,546
	All conditions	Yes	0	0
Beckstead *et al*. [95]	Third instar	Yes	4,015	1,000,994
Estrada *et al*. [93]	All conditions	Yes	2,978	657,929
Wertheim *et al*. [58]	All conditions	Yes	2,280	551,684
Beckstead *et al*. [95]	*Ecr*	No	0	0
Li *et al*. [91]	All conditions	No	0	0

List of all datasets used in this study. The unit of data which we call a dataset is contained in the 'dataset' column. The filtering criteria apply to the microarray data as described in the Materials and methods section. The number of unique genes and functional relationships that a dataset contributes to the integrated network are listed. A '0' indicates that the dataset was not used for integration. There are two examples, Edwards *et al*. [99] (all conditions) and Sorensen *et al*. [96] (all conditions), where the dataset passed the filter but was not used in the integration. This is because all components in these experiments passed the filter criteria, but to remove redundant data, the subcomponent datasets were taken in favor of the dataset defined over the full set of conditions.

We restricted our use of the GO to the category of biological process (GO:BP). Unless specified, we also required any GO:BP annotations to be examined by a human curator as described on the GO website [56]; therefore, the GO evidence codes of IEA, ND (No biological data available), and NR (Not recorded) were removed. Please refer to the Materials and methods section for details on how annotations are handled given the structure of the GO.

### Shared biological processes across datasets

**Figure 1:**
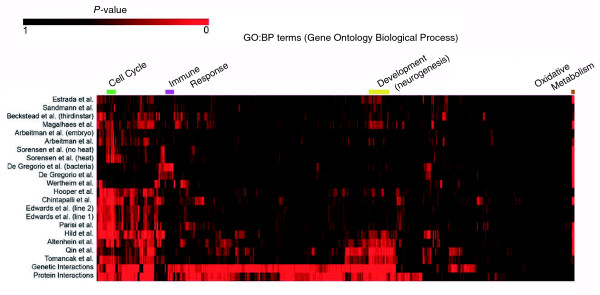
Significant GO:BP terms across datasets. Visualization of how well a dataset connects genes annotated with the same GO:BP term. The dataset names are listed on the left (see Table 1 for citations) and GO:BP terms are listed across the top. All datasets shown are used in the weighted sum (*WS*) integration. From black to red represents the least significant to the most significant GO:BP terms within a dataset as measured through statistically significant coherence (see the Materials and methods section). Both GO:BP terms and datasets were hierarchically clustered and visualized using TM4 MEV [112]. The colored blocks on the top of the figure highlight similar GO:BP terms selected to show different patterns of significance across the datasets. Marked in brown are oxidative metabolism GO:BP terms, which are significant in most MA datasets but absent from the genetic interaction and protein interaction datasets. Marked in green are cell cycle GO:BP terms, which are well represented across most datasets. Marked in yellow are development and neurogenesis GO:BP terms, which are overrepresented in the Magalhaes *et al*. [59] dataset (a microarray experiment on axon guidance). Marked in purple are immune response related GO:BP terms, which are well represented in the DeGregorio *et al*. [57] and Wertheim *et al*. [58] datasets, both of which tested gene expression of immune response.

Understanding the degree of overlap in biological processes amongst the datasets is integral in determining how the information contained in each dataset should be integrated. We explored this overlap by measuring how well a dataset connects genes involved in the same annotated GO:BP. The GI and PPI datasets are each a compendium of all reported interactions, many from largely unbiased screens, that is, Y2H and modifier screens; therefore, we would expect these datasets to provide links between genes across a diverse range of biological processes. On the other hand, individual MA datasets measure gene expression across distinct biological conditions such as time, space, genotype, or stress/treatment. Therefore, we would expect that within each MA dataset, genes with correlated expression profiles will reflect the biological processes that are affected under the experimental conditions. For instance, we expect that genes involved in immune response will show expression changes upon infection with bacteria or fungus, as studied in De Gregorio *et al*. [57]. In order to evaluate the datasets, we first counted the number of gene pairs that were co-annotated with the same GO:BP term. This count was done for each dataset where gene pairs were measured as: statistically significant Pearson correlation coefficients for MAs; all GIs; or all PPIs. The results of performing this test for all GO:BP terms across the GI, PPI (direct assay, high-confidence Y2H, and Y2H are combined in this case), and 20 MA datasets are shown in Figure 1 (see Additional data file 1 for the data used to create Figure 1).

A large number of statistically significant GO:BP terms were revealed across all the datasets, with some terms being nearly ubiquitously significant. In other words, genes annotated with a particular GO:BP term were much more highly connected than expected at random for almost all datasets. The example of cell cycle related GO:BP terms is marked in green in Figure 1. This is a specific example where functional connections between cell cycle-related genes can be strengthened by looking across multiple datasets. Additionally, there are processes that are only found in MA datasets and not in GIs or PPIs; for example, processes involved in oxidative metabolism, namely electron transport and oxidative phosphorylation (Figure 1, marked in brown). Conversely, we also see GO:BP terms that are uniquely significant to a particular dataset. For instance, De Gregorio *et al*. [57] and Wertheim *et al*. [58] performed MA experiments to explore the gene expression responses of flies upon infection with bacteria and fungus, and parasitoid wasps, respectively, and we see that these two datasets are highly significant for immune response GO:BP annotations (Figure 1, marked in purple), while the other MA datasets are largely not well-represented in this class of GO:BP terms. Similarly, Magalhaes *et al*. [59] sampled gene expression related to axon guidance and we see that this dataset is highly significant for developmental biological processes, particularly neurogenesis (Figure 1, marked in yellow). Overall, the GIs and PPIs have the greatest proportion of significant GO:BP terms, while MA datasets vary in the number and kind of GO:BP terms that are statistically significant. Finally, while some GO:BP tend to be common to several of the MA datasets, it is clear that none of the MA datasets provide fully redundant information. This is to be expected given the wide range of biological conditions surveyed in the experiments, and indicates that the data are not strongly biased towards a limited range of biological processes.

These results show that no individual dataset fully represents all biological processes and we see that the datasets both complement and supplement each other, suggesting that integration can be used to more accurately group genes that share biological processes.

### Calculating the likelihood that gene pairs participate in a common biological process

While the GI, PPI, and MA data each provide evidence for gene pair involvement in a common biological process, each type of data has a different measure. GIs and PPIs are reported as Boolean, while the correlations between gene expression profiles in MA experiments are continuous (Pearson correlation coefficient [-1, 1]). We utilized the *LLS *approach, developed by Lee *et al*. [12,28], to convert the gene pair measures from each dataset to a common scale. The *LLS *(Equation 2) reflects how well the relationships in a given dataset agree with GO:BP annotations (see Materials and methods section for details). This approach achieves two important objectives. First, since we are calculating the *LLS *with respect to GO:BP annotation, this score reflects the likelihood that any two genes connected within a dataset share a common biological process. Second, because the *LLS*s for all the classes of data are calculated with respect to the same benchmark set of GO:BP terms, each dataset can now be directly compared.

**Figure 2:**
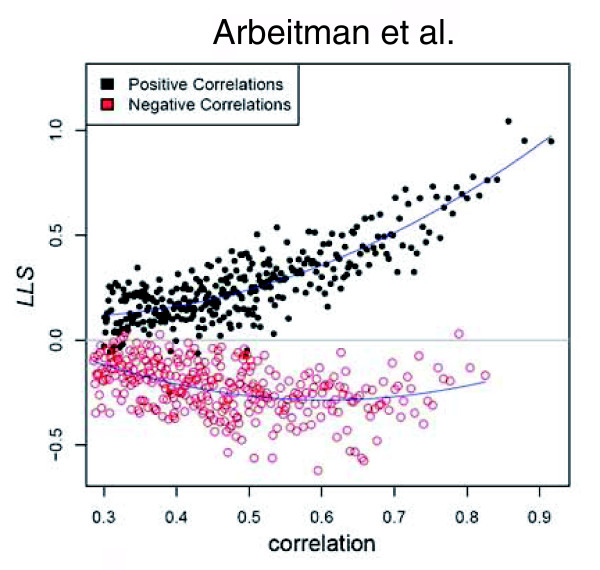
Log-likelihood score calculated for a microarray dataset. The log-likelihood score (*LLS*) compared to the significant correlation coefficients for the Arbeitman *et al*. [61] microarray dataset. Statistically significant correlation coefficients are rank ordered and separated into bins of 1,000 gene pairs. For example, the right-most black dot represents the top 1,000 ranked gene pairs by correlation coefficient. The black dots are positively correlated gene pairs, while the red circles are the absolute value of the negatively correlated expression profiles. The blue line is the polynomial model fit to the data and used to transform all correlation coefficients to *LLS*s.

*LLS*s were calculated for all 24 datasets. We treated all reported GIs as Boolean and then calculated a single *LLS *of 2.661 for the entire dataset. Although the PPI data are reported as Boolean interactions, assay types differ in reliability [60]. We expect direct assay (that is, co-immunoprecipitation, biochemical assay) to be the most reliable, followed by high-confidence Y2H (as defined in Giot *et al*. [54]), then Y2H; therefore, we calculated separate *LLS*s for each class. Our expectations were borne out with a *LLS *of 2.389 for direct assay, 1.045 for high-confidence Y2H, and 0.630 for Y2H. As mentioned, the similarity measures for MA data are continuous correlation coefficients. We expect that gene pairs with the most similar expression profiles will have the highest likelihood of sharing a biological process, and the gene pairs with the least similar expression profiles (coefficient of 0) will have the lowest likelihood of sharing a biological process. Therefore, for each MA dataset, we rank ordered the gene pairs with statistically significant correlation coefficients, divided the ranked list into sequential bins of one thousand, then calculated the *LLS *for each bin. As expected, most MA datasets showed a trend towards increasing *LLS *as correlation values increased. An example can be seen in Figure 2, which reflects this calculation for the Arbeitman *et al*. [61] fly life-cycle timecourse (see Figure S1 at [55] for all additional plots). Interestingly, the most positively correlated and statistically significant gene pairs, in the interval [0.3,1], show a trend of increasing *LLS *with increasing correlation, while the most negatively correlated and statistically significant gene pairs, in the interval [-1,-0.3] (absolute value in Figure 1), show a trend of flat to decreasing *LLS *with more inversely correlated gene pairs. This trend was observed for all the MA datasets. Given the poor performance reflected by the *LLS*s, we removed negatively correlated gene expression profiles from the integration process and only considered positively correlated MA gene pairs. For each of the *LLS *versus positive correlation plots, a polynomial regression was calculated to model the overall trend (blue curve in Figure 2). All pairwise correlation values were then assigned a *LLS *computed from the regressed curve. *LLS*s across all microarray datasets range from 0.1 to 2.3. The *LLS*s calculated for GI, PPI, and MA data indicate that each of these types of data provide evidence for GO:BP annotation shared between gene pairs. We therefore aimed to utilize the *LLS*s with the expectation that, by integrating across all data, we should observe stronger evidence of shared biological processes between two genes than can be detected in individual types of data.

### Integrating the data to construct functional gene networks

Our analysis of the overlap between datasets indicated that, for most biological processes, multiple datasets provided supporting information, but no single dataset provides the preponderance of information. (see the 'Shared biological processes across datasets' section and Figure 1). Based on this observation, we expected that the weighted sum (*WS*) approach, which has been shown to be effective in integrating data in yeast [12,28], worm [16], and mouse [31], would be equally as effective an approach to integrating fly data. In order to test this, we constructed integrated functional networks using the *WS *method developed by Lee *et al*. [12,28]. The *WS *approach mathematically integrates (through weighting) the *LLS*s for gene pairs across the multiple datasets into one measure reflecting our confidence that a gene pair is functionally related.

The *WS *calculation was performed by first rank ordering the *LLS*s for a gene pair, then summing the scores (Equation 4). Included in the *WS *calculation is the parameter, *M*, that down-weights subsequently ranked *LLS*s for a gene pair, where *M *ε 1. Increasing the value of *M *results in greater emphasis being placed on the datasets that provide the greatest likelihood that the members of a gene pair are functionally related. We evaluated the performance of networks constructed with a range of values for the *M *parameter (from 1 to approaching infinity (*M *→ ∞), where *M *→ ∞ effectively only considers the greatest *LLS *for a gene pair). We also tested the naïve approach of summing across all *LLS*s. By varying the values of *M*, we assessed the network's performance on tasks described in more detail below to search for an optimal *M *value.

**Figure 3:**
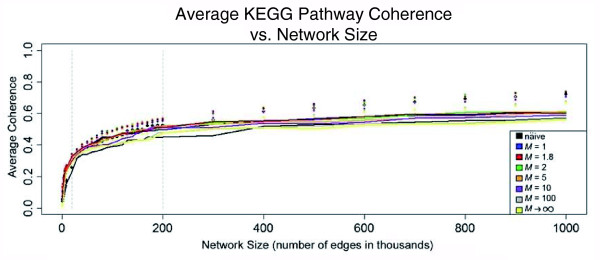
Average KEGG pathway coherence for integration evaluation. The average coherence of 25 KEGG pathways over different weighted sum (*WS*) integrations at increasing network sizes (number of edges). The dots represent the actual measured values averaged over 25 KEGG pathways, while the lines represent the difference between the actual measured values and random coherence at an equivalent network size. The coherence is measured over networks of increasing size up to one million gene pairs. The grey dashed lines mark the network sizes of 20 *K *and 200 *K*, which are the points where the slope (gain in coherence) flattens.

We additionally evaluated the performance of integrated networks with varying network sizes (number of edges in a network). We were interested in the networks' ability to recapitulate known functional relationships between genes reported in the Kyoto Encyclopedia of Genes and Genomes (KEGG) pathways database [62]. We selected the KEGG pathway database for this evaluation since, despite being biased towards biochemical pathways and not entirely independent of GO annotations, it is nevertheless the most appropriate, large, and high-confidence set of annotated functional relationships available for *Drosophila*. Networks were constructed by rank ordering the *WS *scores for all gene pairs and then progressively lowering the threshold on the *WS *score to add edges to the network. Figure 3 shows the performance of the *WS *integration related to network sizes as measured through KEGG pathways coherence, a measure of how tightly a set of genes are connected in a network (see Materials and methods section for details). The dots in Figure 3 represent the average coherence values measured over network size intervals, while the solid lines represent the average coherence values minus the coherence of random sets of genes. The solid lines thus represent the true gain in coherence with increasing network size that is not due to noise. Two important trends are evident. First, the networks constructed with 1 <*M *<< ∞ are more effective at constructing coherent networks than the naïve approach or where *M *→ ∞. Further evaluation revealed an optimized *M *parameter of *M *= 1.8. Second, Figure 3 shows two points at the network sizes of 20 *K *and 200 *K *edges where the slope of the lines flatten. These points reflect the two network sizes that show the greatest KEGG pathway coherence related to network size. We have therefore focused further analysis on 20 *K *and 200 *K *networks constructed using *M *= 1.8. We designate these in the form  to account for both the value of *M *(Equation 4) and the size of network (where the *net size *is in thousands of edges). Both  and  are supplied at [63,64]. Also, the full set of integrated data with over 25 million gene pairs and their associated *WS *scores covering approximately 85% of the protein-coding genes in v5.3 of the *D. melanogaster *genome are supplied at [65].

### Validation of integration

Although data can be integrated, the derived relationships must be vetted. Validation of the integrated gene network data was done in two ways. First, we evaluated how well the integrated network recovered relationships in individual KEGG pathways. Second, we compared the integrated network to networks built from different, individual datasets to test whether integrating the data results in improved performance.

All KEGG pathways containing at least 10 *D. melanogaster *genes were tested against . In total, 63 KEGG pathways were tested. Of these, 59 are statistically significant at a corrected *P*-value < 10^-20 ^as quantitatively assessed using permutation testing and single sample Wilcoxon signed-rank test (see Table S5 at [55] for more details). The number of coherent KEGG pathways and the degree of statistical significance of these pathways provide evidence that the derived functional relationships are biologically meaningful.

**Figure 4:**
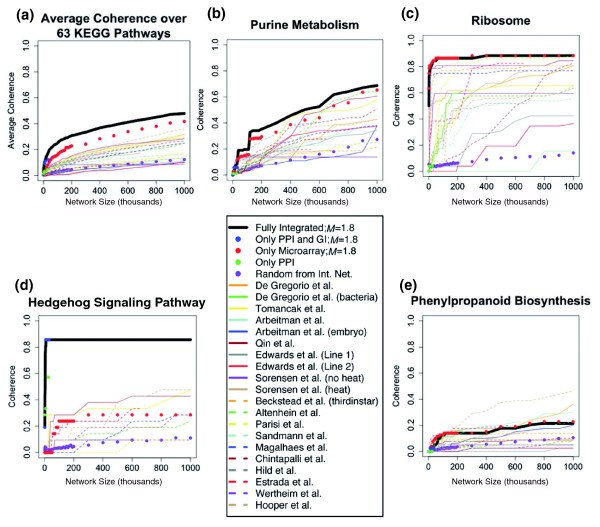
Coherence of types of data and datasets on individual KEGG pathways. Examples of how types of data and individual datasets compare to the fully integrated network as measured through coherence of KEGG pathways [62]. The average coherence of a given dataset is calculated for a set of genes defined by a KEGG pathway at increasing network sizes up to one million edges. **(a) **The average coherence over 63 tested KEGG pathways. The full integration of genetic interactions, protein interactions, and microarray data performs best compared to all other data sources and individual datasets. **(b) **A specific example where the fully integrated network performs better than all other individual datasets and in relation to the 'purine metabolism' KEGG pathways. **(c) **Ribosomal constituents are highly coherent in the microarray data, with many individual microarray datasets performing well. In this instance, not taking into account the genetic interactions and protein interactions performs better than the fully integrated network. **(d) **An example of where the genetic interactions and protein interactions contribute nearly all of the coherent relationships for the 'Hedgehog signaling' KEGG pathway. **(e) **An example of where the integration method performs worse than several individual microarray datasets for the 'phenylpropanoid biosynthesis' KEGG pathway. See Table 1 for citations for the datasets.

We next tested whether the network constructed using integrated data outperforms networks constructed from separate classes of data and individual datasets. We compared the fully integrated gene network to a network built from integrated MA data while ignoring GIs and PPIs, a network built from exclusively GIs and PPIs while ignoring MA data, and a network of only PPIs. We also examined the relative contribution of individual MA datasets. Across the range of network sizes examined (1 *K *to 1,000 *K*; the GI and PPI network and the PPI network have maximum sizes of 32,240 and 25,408, respectively) the average coherence measure (across 63 KEGG pathways) of the fully integrated network was greater than that for the networks based on any subset(s) of data (Figure 4a). This is evident at a network size of 20 *K *where the fully integrated network (GI, PPI, MA) performed the best (area under the curve (AUC) = 0.1020), followed by the GI and PPI network (AUC = 0.0777), and then a step down to the MA only network (AUC = 0.0396). The KEGG pathway coherence for the networks built using the various datasets and summarized as the AUC at network sizes 20 *K *and 200 *K *is provided in Table S6 at [55]. We also see that networks built using the integrated framework outperformed networks based on the individual component datasets. For instance, the integrated MA network performed better (AUC = 0.0396 at 20 *K*) than all networks based on individual MA datasets (maximum AUC = 0.0314 at 20 *K*), and much better than the average individual microarray dataset network (AUC = 0.018 at 20 *K*). In summary, these data indicate that the integrated network performs best in terms of recapitulating known functional relationships across the range of KEGG pathways tested.

We also examined the performance of networks based on the coherence of the various combinations of data with respect to the 63 individual KEGG pathways examined. Given that the fully integrated network performed best when measured against all 63 pathways, we would expect this to be the case for many individual pathways; this was, indeed, the case. For example, the 'purine metabolism' KEGG pathway shows that most of the individual datasets contribute to the coherence and the fully integrated network performs best (Figure 4b). However, it is also clear that the performance of the different datasets varies across different KEGG pathways. For instance, the coherence among genes in the 'Hedgehog signaling' KEGG pathway is based largely on GI and PPI data (Figure 4c), whereas the MA data contribute most of the coherence among genes in the KEGG category 'ribosome' (Figure 4d). There were also cases where networks based on individual datasets outperformed the fully integrated network. This is the case for the 'phenylpropanoid biosynthesis' KEGG pathway, where several individual MA datasets provide greater coherence than the fully integrated network (Figure 4e). While these examples serve to illustrate the ways in which the datasets vary in their performance across specific biological processes, the observed patterns do not fall simply into distinct classes. Plots of all 63 KEGG pathways can be found in Figure S2 at [55] and are summarized in Table S6 at [55]. While the fully integrated network performs best across a wide range of biological processes, the contribution of individual datasets varies across biological processes and there are processes that may be better studied with a subset of data.

### General network properties

**Figure 5:**
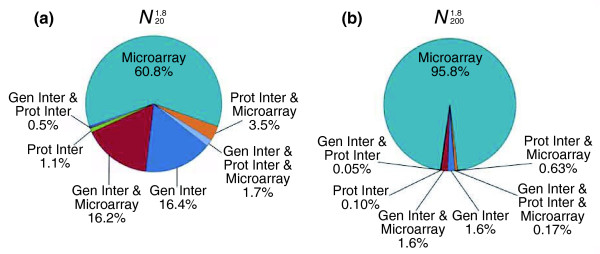
Composition of edges in the integrated networks. Relative contribution of the different types of data to the integrated network of **(a) ** and **(b) **. The teal color represents edges that are drawn solely on microarray data. Dark blue represents edges drawn from genetic interactions only and green from protein interactions only. Orange represents edges drawn from both protein interactions and microarray data. Edges drawn from both genetic interactions and microarray data are in red. Purple represents edges supported by both genetic interactions and protein interactions. Lastly, the light blue represents edges supported by genetic interactions, protein interactions, and microarray data. The colors correspond to the edges in Figure 6.

**Figure 6:**
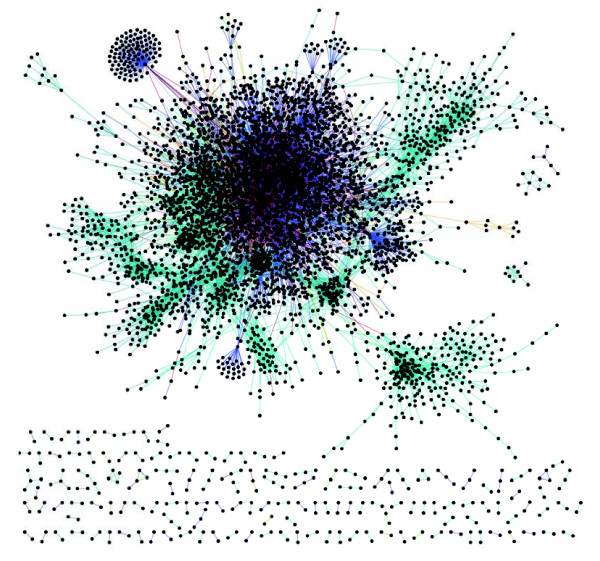
integrated network. Screenshot of  visualized in Cytoscape [66]. The edge colors correspond to Figure 5, where, for example, the teal edges are built from only microarray data and the red edges are built from genetic interaction and microarray data.

 contains 5,021 unique genes and  contains 9,528 unique genes. It should be noted that these networks include any genetic element defined as a 'gene' in FlyBase [46], and consequently includes some elements that have yet to be mapped to the genome (for example, modifier mutations). The inclusion of these elements does not adversely affect the construction of the network; however, it should be kept in mind that while some may represent new genes, many are likely to be alleles of existing genes. Roughly 25% of the genes in  and 13% of the genes in  are of this nature. These genes contribute to 9% of the edges in  and 1.2% of the edges in . The underlying data used to draw an edge in the networks can be any combination of the three types of data (MA, PPI, and GI). In other words, an edge in the network can be based on MA data, MA and GIs, just PPIs, and so on. The composition of the functional relationships between genes can be seen in Figure 5, where the colors in the pie charts correspond to the edge colors in Figure 6, an image of  visualized in Cytoscape [66]. Overall, in , 34.8% of the edges are supported exclusively or partially by GI data, 6.8% are supported exclusively or partially by PPI data, and 82.2% are supported exclusively or partially by MA data. Thus, while the GI and PPI data constitute a very low proportion of the available genomics data, a much greater proportion of these data was used in constructing this network. Specifically, for , 100% of the GI data were used, 5% of the PPI data were used, and 0.004% of the possible edges from MA data were used. As many of the gene pairs used to construct  are supported by PPIs and GIs, these data are also in ; therefore, the edges gained from increasing the size of the network from  to  are from MA data. This can be seen where  has 60.8% of the edges derived solely from MA data and as the network increases to , the number of edges drawn exclusively from MA data increases to 95.8%.

Since the relationships between genes in the integrated network reflect the likelihood that two genes participate in a biological process, we expect that genes involved in the same biological process will cluster together. Manual inspection of  and  reveals both many connections between gene pairs and gene clusters that are consistent with prior knowledge. In order to examine the most prominent examples, we scored and ranked highly interconnected subnetworks within  using Cytoscape [66] and the graph clustering algorithm and visualization tool MCODE [67]. Manual inspection of these subnetworks revealed that the annotated genes within them are largely annotated with common, or closely related, GO:BP terms. (Cytoscape [66] formatted session files, including MCODE clusters, are provided at [63,64]. We have also utilized Java Web Start to make the Cytoscape sessions directly accessible through an internet browser [48].) As an illustration, a subnetwork enriched for genes encoding nuclear ribosomal proteins includes a total of 68 genes, of which 64 encode ribosomal proteins, one encodes a translation initiation factor (*Eukaryotic initiation factor 4A *[FlyBase:FBgn0001942]), and two encode translation elongation factors (*Elongation factor 1β *[FlyBase:FBgn0028737], and *Elongation factor 2b *[FlyBase:FBgn0000559]). A striking feature of the most highly interconnected subnetworks is that they are largely enriched for genes that participate in basic cellular processes such as ribosome biogenesis, the ribosome, proteolysis, mitochondrial electron transport, intracellular protein transport, and cell division, which is consistent with the tight clusters in integrated gene networks in yeast [12,26,28], worm [16], mouse [31,68], and human [27]. Since the functional relationships in the network are based mostly on MA data, this suggests that ubiquitously expressed genes - often referred to as 'housekeeping' genes - are, in fact, coordinately and tightly regulated with distinct expression patterns reflecting their respective biological processes. In addition to expected connections, the network also includes many previously unknown (or previously unnoticed) functional connections, including novel connections between previously studied genes, connections between unannotated and annotated genes, and connections between unannotated genes. For instance, the gene *Receptor of activated protein kinase C 1 *(*Rack1 *[FlyBase:FBgn0020618) is present in the ribosomal proteins cluster already mentioned. Of the 68 genes in this cluster, *Rack1 *is the only gene not annotated with GO:BP terms related to translation. Neither the molecular function ('protein kinase C binding' [GO:0005080]) nor the mutant phenotype (larval lethal and defective oogenesis in germline clones) suggest an involvement in ribosome function [69], but the functional relationships in  suggest a role in the ribosome. This inference is strongly supported by the findings that, in yeast and mammals, highly conserved orthologous proteins are physically associated with the ribosome [70-72]. The preceding examples serve to illustrate that the network can be used to identify functional relationships between groups of interconnected genes as well as the immediate neighbors of any given gene. This in turn provides a means of analyzing new genome-wide datasets with respect to gene function and to infer the annotation of previously unannotated genes. In the following sections we utilize the integrated functional gene network to infer the GO:BP annotations of previously unannotated genes, and explore the use of the network in reanalyzing a genome-wide dataset.

### Inferring biological process gene annotations

Both the  and  networks contain a mixture of annotated and unannotated genes. Specifically, there are 2,544 annotated and 2,477 unannotated genes within , and 3,691 annotated and 5,837 unannotated genes within . A total of 2,673 unique GO:BP terms are associated with the 2,544 annotated genes in , and 2,998 unique GO:BP terms are associated with the 3,691 annotated genes in . Taken together, the functional relationships within the network and the gene-GO:BP annotations provide a means to make *de novo *GO:BP predictions on un- and under-annotated genes. A recent assessment of gene function prediction methods using heterogeneous data sources (a competition among seven groups) demonstrated that reasonably accurate predictions can be made for a metazoan [24]. However, this study also showed that predicting GO:BP terms is more difficult than predicting GO cellular component or molecular function terms - with an average of 21% precision at 20% recall for biological process terms, an average of 32% precision at 20% recall for cellular component terms, and an average of 42% precision at 20% recall for molecular function terms [24]. This assessment provides a useful benchmark for gene function prediction in *Drosophila*. Based on the functional gene network derived from heterogeneous fly data, we explored whether we could make reasonable GO:BP predictions for un- and under-annotated genes.

We calculated the probabilities of gene-GO:BP associations based on the MRF method as described by Letovsky and Kasif [47] (see Materials and methods section). Three key aspects of the network topology and gene-GO:BP term associations are considered: the frequency of a GO:BP term with respect to the tested network; how often genes with the same GO:BP annotation(s) are connected; and the immediate neighbors of the gene whose function is being predicted. Taken in concert, the probability for a gene being annotated with a GO:BP term was calculated using Equation 5. Prediction evaluation was done through tenfold cross-validation. All *D. melanogaster *genes with known GO:BP annotations were divided randomly into ten equally sized groups and GO:BP terms were held-out from one of the ten groups of genes. The *LLS*s were recalculated from scratch using the annotations from the other nine groups. An integrated network was constructed under the *WS *framework (*M *= 1.8) and GO:BP terms were predicted using the MRF method. This procedure was repeated ten times. In the following two sections we use this evaluation to address two questions. First, can we establish a threshold for the prediction posterior probability, denoted *t*_*p*_, that provides reasonable *de novo *predictions? Second, do the predictions from the integrated network outperform predictions made from networks built from individual types of data?

#### Determining prediction thresholds

We first explored the performance of the MRF GO:BP predictions at various thresholds of *t*_*p*_. In order to do this, we calculated the precision and recall of the predicted gene-GO:BP annotations with respect to the held-out gene-GO:BP annotations. It has been observed that measurements of performance on predicted GO terms tend to be quite conservative [24]. This stems from the fact that gene annotation is far from being complete, and the extent to which genes are under-annotated, with as yet undiscovered pleiotropic functions, is not known. This under-annotation will lead to an underestimate of true positives and likely an overestimate of false positives, which will result in a lower measure of precision. Nevertheless, while these performance measures need to be interpreted in light of the fact that they are inherently conservative, they do provide a useful relative measure of performance. Here, a predicted gene-GO:BP annotation was called a true positive if the predicted term matched the held-out term, or the parent or child of the held-out term as defined in the GO. A predicted gene-GO:BP annotation was called a false positive if the predicted term did not match a held-out term on that gene, or a parent or child term. Lastly, a false negative was called for all held-out gene-GO:BP associations where we did not predict a term. In addition to measuring precision and recall in relation to all the held out gene-GO:BP annotations, we also measured the precision and recall with respect to the genes with held-out annotations. In this case we called a gene prediction a true positive if at least one predicted annotation for the gene is a true positive gene-GO:BP prediction. A false positive gene prediction was called if predictions were made for the gene but none were correct. Lastly, a false negative gene prediction was called if the gene had held-out GO:BP terms but we did not make a prediction for the gene.

**Figure 7:**
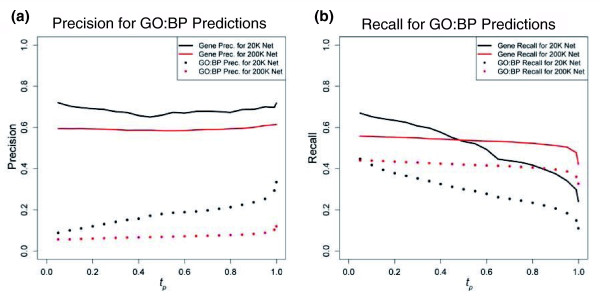
Precision/recall of GO:BP predictions. Precision and recall plots evaluating GO:BP predictions on unannotated *D. melanogaster *genes using the MRF method. The black color reflects predictions made from a network size of 20 *K *and the red color reflects predictions made from a network size of 200 *K*. For the tenfold cross-validation, **(a) **precision and **(b) **recall are shown in relation to the prediction probability (*t*_*p*_). Both precision and recall were measured in relation to all GO:BP predictions and also in relation to the gene (see Materials and methods section for distinction).

Figure 7 shows precision (Figure 7a) and recall (Figure 7b) as a function of *t*_*p*_. These plots show the general trend that increasing *t*_*p *_increases the precision and decreases the recall of gene-GO:BP predictions. In contrast, precision related to gene predictions stays relatively flat over increasing *t*_*p*_. This indicates that, for the predictions made for a gene, at least one has a high likelihood of being true regardless of *t*_*p*_, but the likelihood that any individual GO:BP prediction is true increases with increasing *t*_*p*_. We report a precision for gene-GO:BP predictions of 23% at 20% recall. This is comparable to the average of 21% precision at 20% measured over seven different groups predicting GO:BP annotations for mouse [24]. While it should be noted that there are slight differences in both the input data and the way precision and recall were measured, this comparison serves to illustrate that precision of our predictions is similar to that achieved for another metazoan.

**Figure 8:**
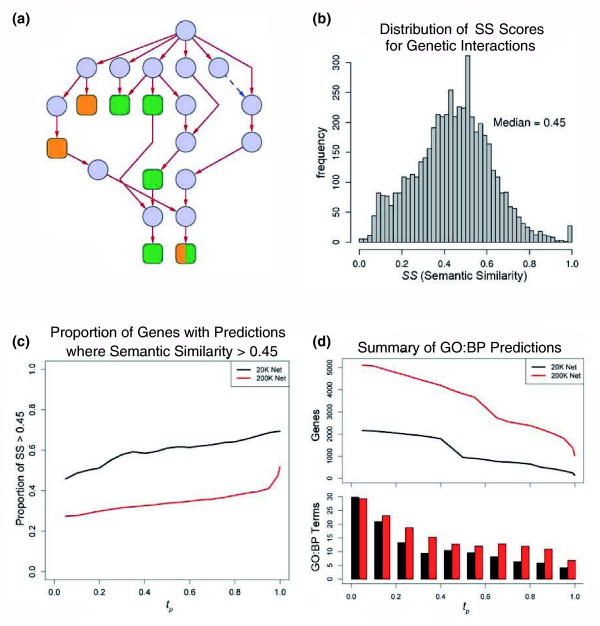
Semantic similarity and GO:BP predictions. Series of plots relating the semantic similarity (*SS*) for tenfold cross-validation to establishing a threshold for the prediction probability, *t*_*p*_. **(a) **An example illustrating the *SS *calculation. The nodes represent GO:BP terms, where the topmost node is the root. The red edges are 'is-a' and the blue, dashed edges are 'part-of' relationships in the ontology. Green nodes represent terms that are known and held-out for one gene, while the orange nodes are examples of predicted terms for the same gene. The half orange, half green node is an example where the predicted term perfectly matches a held-out term. The light blue nodes are the ancestor terms that fall within the path to the root, but are not annotated to either of the genes in this example. The *SS *of (a) is measured to be 0.45 through G-SESAME [73]. **(b) **Also, *SS *= 0.45 is the median *SS *value when measured over all reported and annotated genetic interactions. With respect to the GO:BP predictions, *SS *was measured by comparing the set of predicted terms to the set of held-out terms. **(c,d) **The black color reflects predictions made from a network size of 20 *K *and the red color reflects predictions made from a network size of 200 *K*. (c) The proportion of genes at a given threshold *t*_*p *_that show a *SS *measure of > 0.45. (d) The number of predictions made for both integrated networks,  and . The top plot in (d) shows the total number of genes with at least one prediction in relation to *t*_*p *_and the bottom bar graph shows the average number of GO:BP terms predicted per gene at a given *t*_*p*_.

After establishing the precision and recall for predictions with the integrated networks, we address the first question of establishing a threshold on *t*_*p *_that produces reliable predictions. In order to quantify the similarity between the held-out and predicted annotations in the tenfold cross-validation, we used a measure of semantic similarity (*SS*) and calibrated this measure against a benchmark dataset. In the context of this study, *SS *provides a quantification of the degree of similarity between two sets of GO:BP terms taking into consideration the structure of GO. The measure of *SS *was calculated using the program G-SESAME (Gene Semantic Similarity Analysis and Measurement Tool) developed by Wang *et al*. [73]. The scale ranges from [0,1], where 0 indicates that two sets of GO:BP terms are unrelated, and 1 indicates two sets are the same. As an example, Figure 8a illustrates the overlap of two sets of terms within the structure of the GO where *SS *= 0.45. In order to calibrate this scale with respect to a known benchmark, we examined the distribution of *SS *scores between all pairs of genes with reported GIs (Figure 8b). Since GIs are reliable indicators that two genes function in a common biological process - both experimentally and also shown through the *LLS *- this provided a useful reference set. The median *SS *of gene pairs with reported GIs is 0.45, which we adopted as a reasonable cut-off for our analysis. We then used G-SESAME to measure the *SS *between known GO:BP annotations compared to the predicted GO:BP terms. This was performed for the tenfold cross-validation of both network sizes, 20 *K *and 200 *K*, where *M *= 1.8 over *t*_*p *_∈ [0, 1].

These results can be seen in Figure 8c, where the general trend shows that increasing *t*_*p *_also increases the proportion of genes with predictions that have a *SS *> 0.45 when compared to the held-out annotations for the same set of genes.

Summaries of GO:BP predictions that were made using both  and  are shown in Figure 8d. We can see that at a *t*_*p *_> 0.5, there are an average of 10.5 GO:BP predictions made on 941 genes for  and an average of 12.7 GO:BP predictions made on 3,816 genes for . Extrapolating from the *SS *results shown in Figure 8c, at a *t*_*p *_> 0.5 for , roughly 61% of genes have a set of GO:BP predictions with *SS *> 0.45, so we would expect about 574 genes (941 × 0.61 = 574) to have a set of GO:BP predictions with *SS *> 0.45. See Additional data files 2 and 3 for predictions from both integrated networks.

#### Integrated network increases the performance of predicted annotations

**Figure 9:**
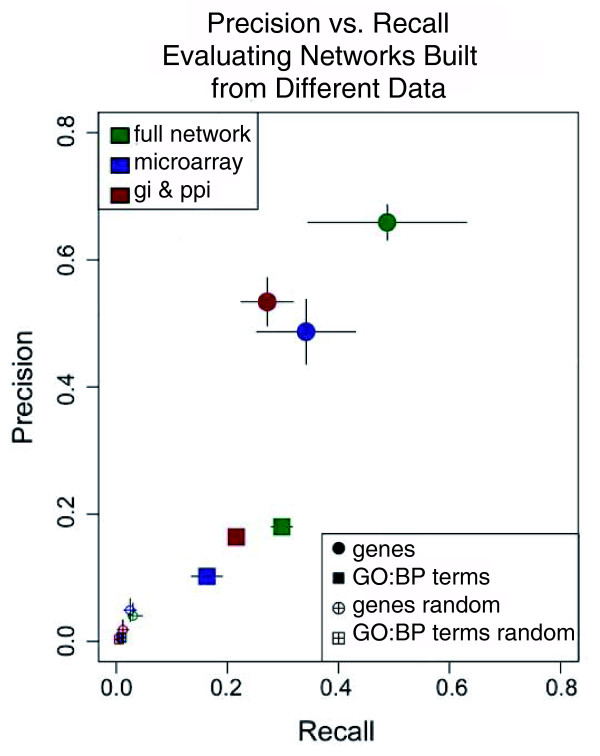
Comparing precision/recall for different data sources. An example of precision and recall calculated on the tenfold cross-validation where the prediction probability is *t*_*p *_≥ 0.5. The colors represent three different networks, all with 20 *K *edges. Blue represents the network built from only microarray data, red represents the network built from only genetic interactions and protein interactions, and green represents the fully integrated network using genetic interactions, protein interactions, and microarray data. The whiskers show the standard deviation of the precision and recall over the tenfold cross-validation. The squares are the precision and recall measures with respect to the GO:BP terms, while the circles are precision and recall as measured for genes (see Materials and methods section for distinction). Predictions of random GO:BP terms are made and the precision and recall are shown as the squares and circles with a plus in the middle.

To address the second question of whether an integrated network built from all three types of data (GI, PPI, and MA) outperformed networks built from individual types of data, we evaluated the predictions in terms of precision and recall with respect to the held-out GO:BP annotations (see Materials and methods section). This was done for three networks built from the following data: fully integrated (GI, PPI and MA); GI and PPI only; and MA only. The integration of the GI and PPI only and MA only data was constructed for networks of 20 *K *and 200 *K *gene pairs using the *WS *framework where *M *= 1.8. When using the fully integrated network, increasing the value of *t*_*p *_resulted in concomitantly increasing precision and decreasing recall. Comparing the results from the three different networks reveals that integrating across all three types of data, on average, outperforms the other two integrated networks (Figure 4; Figure S2 at [55]). The network constructed from GI and PPI data performs better than the network constructed from MA data only for precision and recall with respect to GO:BP terms and precision with respect to genes; however, the MA integration performs better at recalling genes. These results are shown in Figure 9, where the example (*t*_*p *_≥ 0.5 at a network size of 20 *K *edges) is a fair representative of the entire set of evaluations. It should also be noted that we tested the precision and recall of random predictions, where a GO:BP prediction from the MRF method was replaced with a random GO:BP term at the same level in the GO hierarchy. These random predictions performed very poorly and consistently returned less than 1% for both precision and recall. These results demonstrate that the fully integrated network does, in fact, provide more reliable predictions than either of the other networks.

#### Qualitative assessment of GO:BP predictions

In order to provide a qualitative assessment of the GO:BP predictions, we manually inspected the set of predictions made on genes without experimental evidence for any GO:BP annotation. Predictions from  (*t*_*p *_≥ 0.5) resulted in roughly 3,000 gene-GO:BP predictions over 941 unique genes. Of the 941 genes, we excluded 458 that could either not be localized to the v5.3 *D. melanogaster *genome or had at least one known GO:BP annotation (not IEA, NR, or ND). Thus, the set of gene predictions consisted of 1,148 gene-GO:BP predictions over 483 unique genes that could be localized to the genome and did not have any experimental annotation (10% of unannotated *Drosophila *genes).

These predictions were then examined in light of electronically inferred GO:BP terms, molecular function GO and cellular component GO annotation and also an updated version of gene annotation from v5.7 of the *D. melanogaster *genome. We also considered the best non-*Drosophila *sequence matches to the NCBI nr database, along with the respective annotations of these sequences. Over the entire set of 1,148 gene-GO:BP predictions, we found roughly 18% have supporting evidence concordant with our predictions (Additional data file 4). The next two paragraphs provide a few examples of the types of supporting relationships within this 18%.

In our set of predictions, there are several examples of well-studied genes that provide inadvertent cases of well-supported validation. For instance, there are examples of genes whose annotation was not recorded in v5.3 of the *D. melanogaster *genome, such as *Cenp-C *[FlyBase:FBgn0086697] and *crossveinless *[FlyBase:FBgn0000394]. *Cenp-C *is known to be a component of the centromere at mitotic anaphase [74], which we predicted to be involved in 'mitotic sister chromatid segregation' [GO:0000070]. Another example is *crossveinless*, which is known to function in bone morphogenetic protein (BMP) signaling required for wing crossvein development [75,76]. We correctly predicted the GO:BP terms 'imaginal disc-derived wing vein morphogenesis' [GO:0008586], 'regulation of BMP signaling pathway' [GO:0030510], 'torso signaling pathway' [GO:0008293], and 'regulation of transforming growth factor β receptor signaling pathway' [GO:0017015]; however, we also, and potentially erroneously, predicted 'blastoderm segmentation' [GO:0007350] and 'terminal region determination' [GO:0007362].

Further confirmation of prediction quality comes from unannotated genes with additional supporting evidence that is consistent with our predictions. For instance, *CG5525 *[FlyBase:FBgn0032444] was predicted to be involved in 'protein folding' [GO:0006457] where *t*_*p *_= 1. Within the data used from v5.3 of the *D. melanogaster *genome, there was no experimental evidence for any GO:BP terms, but 'protein folding' [GO:0006457] was inferred from electronic annotation and this gene was also annotated with the cellular component GO term 'chaperonin-containing T-complex' [GO:0005832], inferred from sequence similarity. Additionally, the top BLAST hits (default settings) are chaperonin genes from *Culex pipiens *and *Aedes aegypti*. *CG5525 *is an example where the network prediction is consistent with gene function predicted from sequence similarity. As a final example, *Nuf2 *[FlyBase:FBgn0031886] was predicted to be involved in 'M phase' [GO:0000279] where *t*_*p *_= 0.986. From the v5.3 annotations, this gene was inferred through electronic annotation to be involved in 'immune response' [GO:0006955]. However, when checked against the updated annotation of v5.7, *Nuf2 *was annotated with 'chromosome segregation' [GO:0007059], 'mitotic metaphase plate congression' [GO:0007080], and 'mitotic spindle organization and biogenesis' [GO:0007052], all of which are implied from a mutant phenotype. *Nuf2 *is an example where the prediction was validated through experimental evidence that became available after our predictions were made.

Overall, GO:BP predictions have been evaluated using precision/recall and *SS *in tenfold cross-validation. We then used these data to extrapolate the expected number of reasonable predictions that were made using the fully integrated networks. We have also evaluated the predictions qualitatively and shown that roughly 18% have independent evidence that supports the predictions. As a complete analysis, this suggests that the GO:BP predictions are valid.

#### Function prediction on genes with novel sequence features

The GO:BP predictions are based on the functional relationships drawn from the integrated gene networks. The construction of these relationships does not directly take into account any sequence-based information. Traditionally, function prediction methods have relied heavily on sequence and structural similarity [3,4]. As a comparison, we used sequence similarity to infer GO:BP terms for the set of 483 genes for which we have made high-confidence network-based predictions. The translated proteins from these genes were used to search the NCBI nr database using BLASTp (*E*-value < 10^-6^). All BLAST hits to *Drosophila *proteins were removed, matches under 40% identity were removed, then the top 10 hits were taken for each gene. Any associated GO:BP annotations (including IEA, NR, or ND) for the top ten hits were then transferred to the *D. melanogaster *gene. We were able to transfer GO:BP annotations for 224 of the 483 genes. Interestingly, when the GO evidence codes of IEA, ND, and NR were removed, the number of genes with any transferable annotation dropped to 98 of the 483. The *D. melanogaster *genes for which we predicted GO:BP terms using the integrated data appear to be in a class of genes where prediction of biological processes based solely on sequence similarity performs poorly. This is not surprising given the wide scoping meaning of biological process versus sequence features, which often reflect a molecular function, that is, kinase domain or DNA binding domain. Thus, gene prediction utilizing integrated gene networks is a complementary method to make predictions for the class of unannotated genes where traditional function prediction methods perform poorly.

### Interpreting new datasets

Genome-wide functional genomics experiments typically yield lengthy lists of genes that are often difficult to interpret. Common approaches to investigate the biological meaning of these gene lists include GO term enrichment analysis and gene set enrichment analysis (GSEA) (reviewed in [77,78]). Both approaches are dependent on the completeness and quality of the pre-existing reference data: gene annotations in the case of GO term analysis, and gene sets in the case of GSEA. Given that our functional gene network includes previously unannotated genes and clusters together with genes with shared biological processes, we expect that it can be used for improved interpretation of existing and new genome-wide datasets. In order to test this conjecture, we selected a microarray dataset (not used in the construction of the network) and reanalyzed the data with respect to the integrated *Drosophila *gene network. We used data from Teleman *et al*. [79], who examined genes regulated in response to nutrient deprivation in *D. melanogaster *larvae. In particular, we focused on the genes that were found to be significantly differentially expressed (DE) in the muscle tissue of starved larvae.

**Figure 10:**
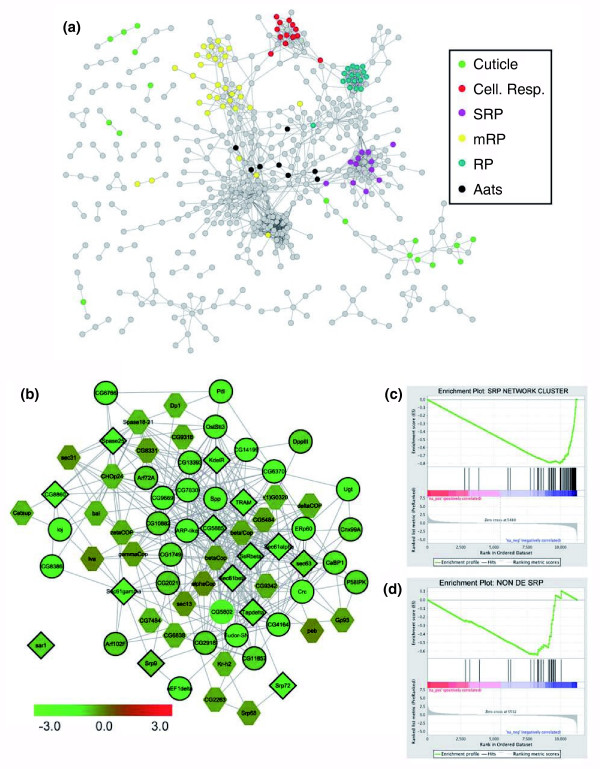
Network analysis in coordination with microarray data. Analysis combing the integrated *Drosophila *gene network and microarray data from Teleman *et al*. [79]. **(a) **The network represents the differentially expressed genes in starved versus fed larval muscle tissue that could also be found in . Several examples of categories of genes listed in Teleman *et al*. are highlighted: cuticle, cellular respiration (Cell. Resp.), signal recognition particle (SRP), mitochondrial ribosomal proteins (mRP), ribosomal proteins (RP), and tRNA synthetases (Aats). The clustering of genes is a result of the integrated network and was done irrespective of the gene expression data from Teleman *et al*. **(b) **The subnetwork is the network built from a seeded set of SRP-related genes as defined by Teleman *et al*. and derived from  (see Materials and methods section for seeded network construction). Gene expression ratios reflect wild-type larval muscle tissue upon starvation over wild-type larval muscle tissue under normal feeding conditions, where green represents genes down-regulated upon starvation and red genes up-regulated upon starvation. All nodes with a dark outline are differentially expressed (DE) genes as defined in Teleman *et al*. The diamond nodes are the seed genes, the circle nodes are genes reported as DE in Teleman *et al*. but not used as seed genes, and the hexagon nodes are genes not reported as DE by Teleman *et al*. The genes in the network in (b) were then treated as a gene set and used as input to GSEA [81]. **(c) **The enrichment plot for all genes in the network in (b). Additionally, we performed an GSEA analysis on the genes in the network in (b) that did not include the seed genes (which corresponds to the set of genes that are circle and hexagon-shaped). **(d) **The enrichment plot for this set of genes showing that the network places together similarly regulated genes that are still significantly enriched even when the set of genes defined in Teleman *et al*. were excluded. See Figure S3 at [55] for more detail on the global performance of gene sets. The gene set representing (d) corresponds to the purple line in Figure S3a at [55].

We first examined whether the network might be used as an aide for classifying DE genes into functional categories. Teleman *et al*. [79] identified 1,943 genes that were statistically DE in larval muscle tissue in response to starvation. Of these, 300 genes were classified according to their annotated functions and are explicitly discussed in the text and figures (referred to here as *DE-categorized*) and the remaining 1,700 genes were not assigned to the categories discussed in the manuscript (referred to here as *DE-uncategorized*). The *DE-categorized *genes were assigned to 16 categories, the prominent ones encompassing carbohydrate metabolism, lipid metabolism, mitochondrial biogenesis and function, cellular translational capacity, and cuticle proteins [79]. In order to visualize the functional connections among all of the DE genes, we mapped them onto  and identified 530 genes sharing 1,536 edges within the network (single gene networks were removed). Inspecting this network revealed three observations (Figure 10a). First, a large number of genes grouped together into distinct clusters, and these clusters are largely concordant with the categories reported in Teleman *et al*. [79] (we highlight a few of the prominent categories in Figure 10a). For instance, of the 20 *DE-categorized *genes in the ribosomal protein category that were found in the network, 19 are tightly clustered (blue in Figure 10a). It should be noted that this was not the case for all categories. For instance, only half of the *DE-categorized *genes in the cuticle protein category were clustered together in the network. Second, the network clusters include *DE-uncategorized *genes interconnected with the *DE-categorized *genes. For instance, a single tightly interconnected subnetwork that includes 11 *DE-categorized *genes in cellular respiration also includes an additional 12 *DE-uncategorized *genes. Third, there is at least one tightly interconnected subnetwork that is composed almost exclusively of *DE-uncategorized *genes. The annotated genes in this subnetwork are enriched for terms related to ribosome biogenesis; however, many of the genes in this subnetwork are unannotated. Thus, the functional gene network revealed that many more DE genes can be grouped into the identified categories and also suggests the existence of at least one additional cluster of genes with the putative function of ribosome biogenesis, which is entirely consistent with the functions studied in Teleman *et al*. [79].

We next examined whether the network could be used to expand the list of genes found to be differentially regulated. To do this, we focused on the set of *DE-categorized *genes reported in Teleman *et al*. [79] as being associated with signal recognition particle (SRP) function. We used the 14 such genes that could be found in the network as a query set to retrieve tightly connected genes from  (see Materials and methods section for details on the search algorithm). This retrieved 56 additional genes selected solely on the connections present in . This network of 70 genes is shown in Figure 10b and the genes are designated as follows: the set of 14 query genes defined in Teleman *et al*. [79] (shown in Figure 10b as diamond nodes), 30 *DE-uncategorized *genes (shown in Figure 10b as circular nodes), and an additional 26 genes that were not determined to be DE in response to starvation [79] (referred to here as *non-DE *genes and shown in Figure 10b as hexagonal nodes). Of the 56 genes added through the integrated network, 18 are annotated as being involved in protein secretion, including the SRP, ER translocon, signal peptidase complex, cargo receptors, and COPI and COPII vesicle components. Interestingly, the annotated set of 18 additional genes largely encode components of the COPI and COPII vesicles (for example, *CG10882 *[FlyBase:FBgn0031408], *Arf72A *[FlyBase:FBgn0000115], *Arf102E *[FlyBase:FBgn0013749], *δCop *[FlyBase:FBgn0028969], *ζCop *[FlyBase:FBgn0040512], *β'Cop *[FlyBase:FBgn0025724], *βCop *[FlyBase:FBgn0008635], *γCop *[FlyBase:FBgn0028968], *Sec13 *[FlyBase:FBgn0024509], *Sec31 *[FlyBase:FBgn0033339], and *αCop *[FlyBase:FBgn0025725]) [80]. (See Additional data file 5 for further annotation information on this cluster of 70 genes.) Using GSEA [81], we tested whether this expanded set of 56 genes was collectively enriched for downregulated genes. Both the full set of 70 genes (Figure 10c) and the subset of 56 (Figure 10d) show a significant enrichment score at a false discovery rate of < 10%. Thus, using the functional gene network, we identified an additional 56 genes that are interconnected in the functional gene network and are collectively significant in GSEA. This example serves to illustrate that the functional gene network can be used effectively to interpret functional genomics datasets. We performed this same analysis for all the categories defined in Teleman *et al*. [79] and consistently found that the gene sets identified using the functional gene network generally performed as well, if not better, than gene sets identified in the original study or those constructed according to GO or KEGG (Figure S3 at [55]).

## Discussion

The focus of this work is to produce a resource that provides the most comprehensive set of experimentally supported functional relationships between fly genes. Thus, we present the first, comprehensive functional gene networks for *D. melanogaster *by integrating experimentally disparate sources of data. The integrated networks are a community resource that benefits researchers in three ways. First, we have distilled a major portion of the extant fly data (over 48 million individual measurements) into functional relationships between genes. The *WS *value of a functional relationship is easily interpretable as the measure of confidence that a gene pair is involved in a shared biological process based on the experimental evidence; however, trying to make sense of the same individual datasets outside the integrative framework is not easily manageable. Second, the functional relationships are built on experimental evidence, which can be easily retrieved to determine the dataset(s) underlying the connection. Third, and as demonstrated in this study, the functional relationships drawn between genes are biologically supported through computational validation. Thus, the networks can be used to derive experimentally testable hypotheses related to gene function.

Understanding the function of every gene in the genome is a central goal of modern biology and integrated networks are another resource that draws a connection from gene to function. To demonstrate the utility of the integrated functional gene networks, we must show that they provide higher quality information than any individual dataset. We have demonstrated this by showing that KEGG pathways are, on average, more coherent within the integrated network compared to any individual dataset or type of data (Figure 4; Table S6 at [55]). We have also shown that edges drawn between gene pairs in the network are consistent with our biological expectation by revealing highly interconnected subnetworks of genes that are consistent with a common biological process. We then used the networks to predict GO:BP terms for un- and under-annotated genes. From these predictions we have shown that the integrated networks outperform individual types of data in both precision and recall, and we can predict GO:BP terms that are semantically similar to known annotation. These observations support the idea that integrated functional gene networks can be used to draw more reliable connections between genes and function. Finally, we showed how the integrated gene network can aide in the analysis of microarray data to uncover relationships that would have been missed without the network.

Additionally, we have shown that there is a class of genes where sequence similarity performs poorly for predicting GO:BP terms. Since sequence information is not included in the construction of the integrated functional gene networks, these networks provide another source of confident relationships that can be used to predict biological processes on this class of genes. Function prediction using gene networks complements sequence-based prediction methods. Although we only discuss the most confident GO:BP predictions for 483 genes, we also make predictions that cover more levels in the GO hierarchy and predictions for genes with already known and experimentally supported annotations. These predictions constitute the first genome-scale attempt to use an integrated set of experimental data to make biological process predictions for *D. melanogaster *genes. These predictions are another source of data to aide in identifying the associated biological process(es) of the one-third of *D. melanogaster *protein-coding genes that are currently unannotated.

The functional gene networks are a resource for exploring functional relationships among genes at both the local and global levels. The network sizes 20 *K *and 200 *K *were selected to maximize the number of connected genes that are involved in the same biological process while minimizing the overall number of edges.  is restricted to the most highly supported functional relationships at the expense of including fewer genes and edges. Consequently, users interested in exploring high confidence relationships including specific genes of interest are advised to query  first. On the other hand, the  has a lower threshold that allows for an increased number of genes and connections to be made that are heavily based on microarray data. Thus,  is useful for exploring functional relationships at a more global level supported by gene expression data, as well as identifying relationships between genes that may not be present in .

The integrated networks built from fly data tend to perform well at drawing connections between genes involved in core biological processes and components, such as cell cycle, catabolic processes, the ribosome, and the proteasome. This same trend also holds in the integrated networks built from yeast [12,26,28], worm [16], mouse [31,68], and human [27] data. Issues with repeatability and false positive rate have been raised with genome-scale data, particularly with microarray [82] and yeast-two-hybrid [60,83] assays. Integrative methods mitigate the effect of one data source determining a connection between a gene pair by requiring multiple independent datasets to support the relationship between two genes. Finding core biological processes consistently clustered together across different species, which are derived from different experimental datasets, instills confidence that the relationships are both biologically real and computationally detectable.

Integrative methods are not without their biases. Annotation of genes with GO terms are biased towards well-characterized genes and well-studied processes. For example, 'eye morphogenesis' [GO:0048592] is a widely studied process in *Drosophila *and is associated with over 200 genes, while 'muscle morphogenesis' [GO:0048644], which is at the same level in the GO hierarchy, is annotated to only four genes. Though the number of genes involved in eye or muscle morphogenesis are not expected to be equal, it is likely they would be on par with each other. Certainly we expect there to be more than four genes involved in muscle morphogenesis. Most integration methods, including the one implemented here, require a gold-standard set of comprehensive and biologically validated gene-gene pairs. Genes sharing GO annotation terms have been used as this gold-standard and the biases reflected in annotation will thus be reflected in the final product of data integration methods. Though some biological processes will certainly be underrepresented, integrative methods have been highly productive in constructing networks that both capture the current state of biological knowledge and expand upon this knowledge by drawing connections between genes of unknown function.

Clearly, the quality, scope, and types of experimental data used are key factors in the integrative framework, and incorporating new data, as well as refining the selection of input data, offers the opportunity to improve and tune future networks. This study focuses on producing a comprehensive global functional gene network using available GI, PPI, and MA datasets for *Drosophila*. These datasets were selected based on their ability to connect genes that are involved in the same biological process. Overall, the extant GI data provided the greatest likelihood of gene pairs being functionally related, followed closely by direct assay PPI and the most highly correlated gene pairs within several MA datasets (indicated by the calculated *LLS*). The *LLS*s for MA data drop as the correlation coefficients within the datasets drop, but the reported values are commensurate with high-confidence Y2H and Y2H PPIs. Thus, while these classes of data did not contribute equally, all three provide high quality information used in constructing the global integrated networks. However, there are many datasets available that were not incorporated into the current version of the networks. There are several reasons for this. First, we tested the usefulness of fluorescent *in situ *hybridizations [84] and transcription factor binding sites [85,86] as input data, but these data did not meet the evaluation criteria under the *LLS *framework. Second, there are datasets, such as RNA interference screens [87], that are not easily translated into a measure that can be used under the *LLS *framework. Third, this study focuses on experimentally supported datasets; therefore, computational methods to relate genes [88-90] were ignored. Better utilization of these data sources will likely contribute to increased quality of functional relationships assigned between genes. Additionally, the ongoing modENCODE [44] projects promise an unprecedented increase in high-resolution functional genomics data. Functional gene networks offers one route to help interpret these forthcoming data. On the other hand, we do note that networks constructed using subsets of the data can outperform the global network in identifying relationships among genes in specific KEGG pathways (Figure 4e). Thus, refinement of the current framework, using only selected subsets of the available data, should make it possible to build networks more representative of specific biological processes. Building integrated networks in relation to a particular biological process would likely yield functional relationships more closely related to the specified biological process.

## Conclusions

We have integrated heterogeneous datasets to produce the first comprehensive functional gene network in *D. melanogaster*. We have shown that the functional relationships between genes are highly consistent with KEGG pathways and use these results to construct the two networks  and . We have demonstrated that edges drawn between gene pairs are consistent with our biological expectation by revealing highly interconnected subnetworks of genes that are nearly completely consistent with a common biological process. We also show how the network can be used to enhance the interpretation of microarray data by both discovering clusters of genes that are co-regulated and identifying candidate unannotated genes tightly coordinated with a known and co-regulated biological process. The full set of integrated data and networks built from these data ( and ) are made available. We also provide GO:BP predictions for 2,154 genes in  and for 5,107 genes in . This community resource can be accessed online [48].

## Materials and methods

### Data acquisition, cleaning, normalization, filtering

#### Genetic interactions

GIs were downloaded as a pre-computed file from FlyBase, version FB2007_02 [46]. Interactions containing a gene not belonging to *D. melanogaster *were removed (that is, transgenic construct from *D. simulans*). All reported interactions (6,941) were given the same weight, a value of 1.

#### Protein-protein interactions

All PPIs - *D. melanogaster*-specific where possible - were downloaded from the following databases: BIND [49], DIP (version Dmela20071007) [50], DroID (September 2007) [51], BioGRID (version 2.0.32) [52], IntAct (September 2007) [53]. The varying protein IDs across all datasets were mapped to v5.3 FlyBase gene identifiers. Any IDs that did not unambiguously map to a single FlyBase gene ID were removed. The union of reported interactions across all the datasets was taken. The experimental method used to detect an interaction was also considered. If a reported interaction was detected through multiple experimental methods, the most reliable method was ascribed to the interaction. The order for reliability is as follows: direct assays (that is, co-immunoprecipitation, biochemical assay) > high-confidence Y2H (high-confidence as reported in Giot *et al*. [54]) > Y2H. In total, there were 25,408 reported PPIs among pairs of *D. melanogaster *proteins. These include 1,234 determined by direct assays and 24,408 Y2H interactions. The Y2H assays were subdivided into 4,590 high-confidence interactions and 19,584 positive interactions.

#### Microarray gene expression

The following raw MA datasets were downloaded from Gene Expression Omnibus (GEO): [GEO:GSE94] [61], [GEO:GSE541] [91], [GEO:GSE442] [92], [GEO:GSE3854] [93], [GEO:GSE5430] [94], [GEO:GSE3057] [95], [GEO:GSE3069] [95], [GEO:GSE5147] [96], [GEO:GSE695] [97], [GEO:GSE3257] [98], [GEO:GSE5404] [99], [GEO:GSE6515] [59], [GEO:GSE6186] [100]; [ArrayExpress:E-TABM-57] [101], [ArrayExpress:E-MAXD-6] [58]; and supplemental pages De Gregorio *et al*. [57], Chintapalli *et al*. [102], and Tomancak *et al*. [103]. These data used two distinct platforms; two channel cDNA or oligonucleotide spotted arrays, and single channel Affymetrix short oligonucleotide arrays. All data normalizations were performed in the R statistical programming environment [104]. The datasets selected were required to have at least five conditions to make reliable correlation measures. We also did not use any datasets that were *Drosophila *cell lines.

Two channel experiments were normalized using local regression within the OLIN package [105]. OLIN was run with default parameters, scaling turned on, and flagged spots were ignored for any calculations. The results of the full OLIN normalization are log-transformed ratio values for each gene on each individual MA slide.

The Affymetrix arrays were normalized using the Affy [106] and GCRMA [107] R packages. Affinities for all oligonucleotide sequences were calculated and the 'fullmodel' GCRMA normalization was run, resulting in log-transformed expression values for each probe set on each array.

All spots or probe sets were mapped to the v5.3 *D. melanogaster *genome assembly and annotation. Genome sequence files were downloaded from FlyBase under the FB2007_02 release [46]. Primer-based platforms required two rounds of BLAST; one round to match the primers to the genome (BLASTn; *E*-value < 10^-2^) and the second round to match the amplicon product to the genome (BLASTn; *E*-value < 10^-6^). Physical coordinates from the forward and reverse primers were checked for strandedness and to make sure the PCR product would be under 1,000 nucleotides. The segment of DNA between the forward and reverse primers (including the primers) was taken as the amplicon product for that primer pair and searched back against the genome to ensure the amplicon did not align to any other region outside the intended segment, potentially leading to cross-hybridization. cDNA-based arrays required the cDNA sequence be aligned against the genome to test for potential cross-hybridization. Any amplicons or cDNAs with a second best BLAST hit with 80% sequence identity were flagged and removed. Unique BLAST hits mapping to exons of v5.3 annotated genes were assigned the corresponding FlyBase gene ID, otherwise the spot was flagged and removed.

Sequence files for both Affymetrix *Drosophila *array platforms (versions 1 and 2) were downloaded from the Affymetrix website [108]. They contain a unique sequence for each probe set, which is searched (BLASTn; *E*-value < 10^-6^) against the genome to test for potential cross-hybridization. A segment of DNA associated with a probe set was assigned a v5.3 FlyBase gene ID if the BLAST result showed a putative hit to at least one or part of one exon from one gene. A probe set was not assigned a gene ID and flagged if the BLAST result was ambiguous, meaning the second best BLAST hit was greater than 80% sequence identity, or the query sequence did not hit at least one exon.

For either MA platform, gene expression profiles were constructed using the calculated expression values for a gene across the tested conditions. If a gene expression profile had greater than 25% absent/removed expression values, that gene's profile was removed, otherwise missing values were inferred using KNNimpute [109].

We defined an MA dataset to be the full, published unit of data, and, where possible, datasets were additionally defined as the subcomponents of the published dataset. For example, the Arbeitman *et al*. [61] study contains six datasets; all published conditions, embryo, larva, pupa, adult male, and adult female. See Table 1 for the breakdown of all datasets.

Gene expression profiles that did not change over the course of a dataset - referred to as 'flat' - were filtered out. This was done on a gene by gene basis by taking the difference between the maximum and minimum expression values across all conditions in one dataset. For the Affymetrix platform, if the difference between the maximum and minimum expression values was less than 50, then that gene and corresponding expression profile was removed. For the two channel experiments, if the difference between the maximum and minimum log ratio value was less than.5, then the gene and corresponding expression profile was removed.

#### Genome annotations: Gene Ontology terms

The count of genes annotated for the organisms discussed in the introduction were downloaded from the GO website [56]. The annotation counts were limited to the biological process component of the GO. Additionally, the evidence codes IEA, ND, and NR were ignored.

Specific to *Drosophila*, gene annotations for GO:BP terms were taken from the FB2007_02 version of FlyBase [46]. These data provide a mapping from a FlyBase gene ID to the GO:BP term ID(s). GO:BP terms with the following evidence codes were removed: IEA, ND, and NR. The structure of the GO is a directed acyclic graph, meaning each term has a parent term(s) (the root term is the only exception) and each term potentially has a child term(s). As described in Lord *et al*. [110], a connection was drawn in the ontology for the link types 'is-a' and 'part-of', then each gene was propagated from its annotated position on the GO to the root. Thus, the number of genes associated with any particular term, *t*_*i*_, in the GO includes the genes annotated to *t*_*i *_and additionally subsumes any genes that are annotated to the child term(s) of *t*_*i*_.

#### Additional data

It should be noted that we also evaluated two additional, potential data sources, which include matches to transcription factor binding sites [85,86] and fluorescent *in situ *hybridizations [84]; however, these data were not included as they did not meet our evaluation criteria (data not shown).

#### Microarray profile correlation, statistical significance

In total, 34 MA gene expression datasets were collected, normalized, and filtered. We define these 34 datasets as *D *= {*D*_1_, *D*_2_,...,*D*_34_}. The Pearson correlation coefficient was calculated for all gene pairs in a dataset *D*_*i *_∈ *D*, For *n *genes in *D*_*i *_= {*g*_1_, *g*_2_,...,*g*_*n*_}, each *g*_*j *_∈ *D*_*i *_is a vector of expression values  across *m *conditions. The Pearson correlation coefficient between *g*_*x*_, *g*_*y *_∈ *D*_*i*_, where 1 ≤ *x *≤ *n *and 1 ≤ *y *≤ *n *was calculated as:

(1)

Calculating the correlation between all *g*_*x*_, *g*_*y *_∈ *D*_*i *_results in a distribution of correlation values. Since the majority of correlations do not reflect a functional linear relationships between two genes, only statistically significant correlations were used. Significance of the correlations were assessed through permutation testing. Within each condition of a particular dataset, gene expression values were shuffled, thus randomizing the correlation measures for each gene. From the shuffled data, 20% of the genes were selected at random and the pairwise Pearson correlation coefficient calculated for this subset of genes. This process was then repeated five times to create a stable empirical null distribution of correlation coefficients. Any correlation coefficients with a *P*-value < 0.01 on the two-tail null distribution - corresponding to positive and negative correlation values - were considered for further analysis.

### Calculating significant biological processes across datasets

A total of 22 individual datasets were tested for over-representation of GO:BP terms (details on the GO:BP terms discussed above): all reported GIs; all reported PPIs (direct assay, high-confidence Y2H, and Y2H combined); and for each of the 20 MA datasets used, gene pairs with significant coexpression correlations as defined in the previous section. (Methods to arrive at 20 MA datasets are discussed below in the 'Integration' section.) For each individual dataset, the number of gene pairs annotated to the same GO:BP term were counted. GO:BP terms were only considered if they were annotated to at least 10 and less than 300 *D. melanogaster *genes. The lower cutoff of 10 genes was set in order to calculate reliable statistics and the upper cutoff of 300 was set to not bias the analysis to highly annotated terms. The cutoff of 300 was determined by the information content (*IC*) measured over all GO:BP terms meeting the criteria mentioned in the previous paragraph. The *IC *for *t*_*i *_is calculated as *IC*(*t*_*i*_) = ln(*P*(*t*_*i*_)), where *P*(*t*_*i*_) is the probability that *t*_*i *_is annotated to a gene. *P*(*t*_*i*_) is calculated by finding the fraction of times *t*_*i *_is annotated to a gene compared to the total number of possible annotations. The total number of possible annotations is the count of genes annotated at the root, since the root term subsumes all gene annotations. A qualitative assessment of *IC *measures on GO:BP terms showed a reasonable cutoff corresponding to 300 annotations.

Each GO:BP term used in this analysis has an associated number of *x *genes. To test the significance of a particular GO:BP term within a particular dataset (Figure 1), an empirical null distribution was constructed. For each GO:BP term with *x *associated genes, a random set of *x *genes was selected from the dataset being analyzed, and the number of connections between this set of *x *random genes was determined. This procedure was repeated 100 times. In all cases the counts were normally distributed. Significance of the number of connections between the *x *genes tested was performed through a right-tailed, single-sample *t*-test. This resulted in a matrix of 22 datasets by 1,133 GO:BP terms, where the values in the cells of the matrix are *P*-values. This matrix was hierarchically clustered on both dimensions using TM4 MEV [111,112] with average linkage and Euclidean distance. Visualization of the clustered matrix was also done in TM4 MEV.

### Integration methods

#### Log-likelihood score

The general procedure for integrating gene-gene relationships across all datasets was adapted from Lee *et al*. [12,28]. Datasets and the functional relationships drawn between two genes were scored in relation to GO:BP annotation, where the annotations met the same criteria as mentioned in the previous section. The *LLS *was calculated for each dataset as follows (we will use the same notation as Lee *et al*. [12,28]; in particular ~ denotes 'not'):

(2)

*D *represents a dataset of gene pairs and can be PPI, GI, or MA. *I *represents the set of gene pairs that were annotated and shared at least one GO:BP term, while gene pairs in ~ *I *were annotated, but there was no overlap between the GO:BP terms annotated to individual genes in a pair. Both *I *and ~ *I *are counts taken across all genes in the v5.3 *D. melanogaster *genome. *P*(*I*) is the probability of a gene pair sharing at least one GO:BP annotation, and *P*(~ *I*) is the complement. The probability of finding an annotated gene pair sharing at least one GO:BP term restricted to the gene pairs within dataset *D *is *P*(*I*|*D*), and *P*(~ *I*|*D*) is the complement. In the case of MA data, *D *represents the dataset after being filtered for significant correlation values and removing 'flat' expression profiles.

#### *LLS *for genetic interactions

A *LLS *was calculated for the entire GI dataset. Each reported gene pair was weighted equally; therefore, a gene pair within the GI dataset was assigned a *LLS *score calculated from the entire dataset, where *LLS *= 2.661.

#### *LLS *for protein-protein interactions

The PPI data were separated into three subsets reflecting the expected reliability of the experimental methods to detect interacting proteins. A *LLS *was then calculated for each subset. Protein pairs within a subset were assigned their respective *LLS*s. The first class of PPIs reflected interactions reported in a Y2H assay, where *LLS *= 0.630. The second class reflected interactions defined as high-confidence Y2H, where *LLS *= 1.045. The most confident class of experimental techniques (noted 'direct assay') included co-immunoprecipitation, affinity methods, biochemical assays, and mass spectrometry, where *LLS *= 2.389.

#### *LLS *for microarray datasets

As described in Lee *et al*. [12,28], gene pairs from each individual MA dataset (filtered on significant correlations and 'flat' profiles) were first ordered according to their correlation coefficients and then separated into bins of 1,000 gene pairs, where the first bin contains the most significant positively correlated gene pairs. A *LLS *was then calculated for each bin and plotted against the mean correlation value  for bin *i *(Figure 2). From this plot, we fit the polynomial equation , using the lm() function in R. A separate curve was fit for both positively and negatively correlated data. Every point along the curve for a positive correlation was greater than a *LLS *of 0, while every curve fit to the negative correlations had at least some portion that fell below a *LLS *of 0. Therefore, only the significant positively correlated data were considered in evaluating each MA dataset. From all fit curves, a measure of the fraction of variance explained by the model was calculated as:

(3)

where *f*_*i *_is the *i*^*th *^fitted value of the model, *y*_*i *_is the fitted value plus the residuals for the *i*^*th *^bin, and  is the average of *y*_*i *_over all *i *bins. Additionally, the value for *r*^2 ^was adjusted for the number of coefficients in the model. Datasets that had an adjusted *r*^2 ^< 0.5 were removed from further analysis. Also, datasets were required to have a positive linear trend. After applying these criteria to all MA datasets, 20 of the 34 passed and were used in this study, whereas 14 of the 34 did not meet these criteria and were removed (Table 1; Figure S1 at [55] for all datasets). In two cases (Sorensen *et al*. [96] and Edwards *et al*. [99]), all datasets related to one experiment passed the above criteria. To remove the redundancy with these two cases, the datasets constituting the subcomponents of the experiment were chosen over the full set of conditions. Specifically, the Sorensen *et al*. [96] control timecourse and heat-shocked timecourse were used and the dataset consisting of all conditions was not used. Within the Edwards *et al*. [99] datasets, two lines of flies were tested, so line 1 and line 2 were used and the full set of conditions was not used.

The positively correlated gene pairs in the 20 datasets passing the above criteria were rescored and assigned a *LLS *according to the fit polynomial equation. This rescoring transformed a gene pair's correlation coefficient into a *LLS*.

#### Weighted sum

The weighted sum (*WS*) was adapted from Lee *et al*. [12,28] and was calculated as follows:

(4)

*LLS *values for a gene pair across all *k *datasets were ordered from largest to smallest *LLS*_*i *_≥ *LLS*_*i*+1_, ∀*i*; 0 ≤ *i *≤ *k *1, *M *is a free parameter and can be adjusted to increase or decrease the contribution of subsequently ranked *LLS*s. It should be noted that ignoring the denominator (*i*·*M*) and simply summing all *LLS*s across the *k *datasets is akin to a naïve Bayesian integration. This assumes uniform priors on each of the *k *datasets. Although, this method of integration is not completely Bayesian as the values being summed are *LLS*s and not probabilities. The opposite of ignoring the denominator is to set *M *→ ∞. This causes the *WS *calculation to consider only the *0*^*th *^ranked *LLS *(that is, *WS *= *LLS*_0_). To test a range of integration scores, *WS *calculations were made for all gene pairs where *M *∈ {1,2,5,10,100}, *M *→ ∞, and also for the naïve method. These seven *WS *calculations were selected to cover a range of different weighting schemes.

The KEGG pathways were used to validate functional relationships in the integrated network [113]. To test the overlap between KEGG and GO, we compared gene-gene associations derived from KEGG pathways and the set of GO:BP annotated gene pairs used in our analysis. This comparison revealed that roughly a quarter of the gene pairs from KEGG pathways are also present as gene pairs in GO:BP.

Gene IDs for each KEGG pathway were mapped to the v5.3 genome annotation. The genes in each pathway were tested against a network through the measure of coherence. The network is a graph and can be defined as *G*⟨*V*, *E*⟩ with *V *vertices (genes) and *E *edges (functional relationships). The set of KEGG pathways is defined as *K *= {*K*_1_, *K*_2_,...,*K*_*n*_}, where *K*_*i *_is the set of genes defined by KEGG pathway *K*_*i*_. The greatest connected component for *K*_*i*_, noted , was determined by the greatest number of genes in *K*_*i *_present and creating a connected component in *G*⟨*V*, *E*⟩. The coherence for *K*_*i *_was then calculated as . Twenty-five pathways were selected to evaluate the *WS *integrated networks (Figure 3; the 25 pathways are marked with asterisks in Table S5 at [55]). The 25 KEGG pathways were selected because they consistently showed the highest coherence amongst all the KEGG pathways tested.

The scores for each of the seven *WS *calculations were rank ordered, then networks were built starting from the top 1,000 scoring gene pairs in increasing intervals to networks of one million edges. The average coherence of the 25 pathways over each of the size intervals was measured (Figure 3). The curves in Figure 3 were then used to determine the smallest network size that provides a high overall coherence across KEGG pathways, since the average coherence varies as a function of the size of the network. We identify the points on the curve where the gain in average coherence flattens as the size of the network increases. These points of the curves occur at network sizes of 20 *K *and 200 *K*. These two network sizes are used throughout the rest of this study.

After establishing the network sizes, we aimed to optimize the *M *parameter in the *WS *score to provide the greatest average KEGG pathway coherence. Since most of the coherence was gained by the network size of 200 *K *gene pairs, this network was used to evaluate seven *WS *integration schemes. This was done by measuring the AUC. Large gains of KEGG pathway coherence in the smaller sized networks results in a higher AUC, while slow or little gain in coherence results in a low AUC. Thus, the AUC (Figure 3) is a means of assessing how well a *WS *integration method recovers KEGG pathway relationships. By iteratively testing networks built with increasing *M *values from 1, we determined the *WS *integration where *M *= 1.8 maximized the AUC for the network size of 200 *K *edges.

All KEGG pathways having at least ten *D. melanogaster *genes were tested individually against the *WS *network, where *M *= 1.8 at a size of 200 *K *edges. In total, 63 pathways were tested. Statistically significant coherence measures were evaluated through permutation testing; an empirical null distribution of coherence values was calculated by randomly sampling 1,000 times a set of genes equivalent to |*K*_*i*_|. A single-sample Wilcoxon ranked-sum statistic was used to measure the significance of *K*_*i *_when compared to the null distribution. *P*-values were adjusted using a Bonferroni correction.

### Markov random field method to predict GO:BP

We employed the MRF method implemented by Letovsky and Kasif [47] to predict gene function utilizing an integrated network and known GO:BP terms (excluding IEA, ND, and NR evidence codes). The probability for a gene being annotated with a GO:BP term can be calculated as follows (note that the equations are taken from Letovsky and Kasif [47] and further detail can be found in their manuscript):

(5)

where *L*_*i*, *t *_is a Boolean random variable dependent on gene *i *and term *t*, *N*_*i *_is the number of genes directly adjacent to *i*, and *k*_*i*, *t *_is the number of genes directly adjacent to *i *that are annotated with term *t*. The authors also make the assumption that the degree distribution of nodes labeled with *t *is not significantly different than the overall degree distribution. While this assumption does not hold for all terms *t *in our study, it does for the majority; therefore, we also make this assumption. Ultimately, the authors develop the probabilistic neighborhood function:

(6)

where *f*_*t *_is the frequency of term *t *in the network, *p*_0 _is the probability that any given gene in the network annotated with term *t *is NOT connected to another gene annotated with term *t*, while *p*_1 _is the probability that any given gene in the network annotated with term *t *IS connected to another gene annotated with term *t*. *λ *can be described as the ratio of the weighted frequency of the presence of term *t *annotated to the neighbors of gene *i *over the weighed frequency of the neighbor genes not annotated with term *t*. The ratio relies on the binomial distribution . The MRF method produces a probability for a gene by GO:BP term basis and was run on the networks of size 20 *K *and 200 *K*.

#### Prediction evaluation (precision/recall)

The GO:BP predictions were evaluated using tenfold cross-validation. All genes annotated with GO:BP terms were randomly divided into ten equal sets, *G *= {*G*_1_, *G*_2_,...,*G*_10_}. The following methods are performed for each of the ten sets in *G*. The annotations for all the genes in set *G*_*n *_(where *n *= {1, ..., 10}) were masked from their corresponding genes. The *LLS *and *WS *integration, where *M *= 1.8, were recalculated for each dataset. Note that just the annotations are removed from the set of genes, but the genes remain in the analysis. The newly calculated *WS *relationships were rank ordered and networks with the top 20 *K *values and 200 *K *values were built. These two networks along with the GO:BP annotations from sets {*G*_1_, ..., *G*_10_}-{*G*_*n*_} were then used as input to the MRF prediction method. Predictions were made on all genes in the network and measures can be used to evaluate the performance of predictions in relation to the held-out annotations for *G*_*n*_.

Two methods were used to evaluate the GO:BP predictions made on the genes in *G*_*n*_. First, the precision () and recall () were calculated with respect to GO:BP terms and also with respect to the genes (*tp *= true positives, *fp *= false positive, and *fn *= false negative). The second method measured the semantic similarity (*SS*) between the known set of annotations for a gene and the predicted terms for that gene.

Precision and recall with respect to the GO:BP terms were calculated as follows. A true positive prediction was called if the predicted term exactly matched a known, held-out term, or the known term's parent(s), or the known term's child(ren) (± 1 level in the GO with respect to one GO term). A false positive was called if the predicted term did not match a known, held-out term or a parent or child of the known term. A false negative was called for any known, held-out annotation not called a true positive. It should be noted that we also tested a more stringent criterion of requiring predictions to exactly match known GO:BP terms and a less stringent criterion where predictions can match ± 2 levels in the GO hierarchy. The evaluation method we used is a fair balance between the more and less stringent criteria and the precision/recall values followed the same trends for each of the three tested criteria.

A measure of precision and recall was also calculated in relation to the gene. Extrapolated from the evaluation methods of GO:BP terms, we counted a true positive gene prediction if a gene had at least one true positive GO:BP term prediction. In other words, a true positive gene was called if the intersection between previously known, held-out terms and predicted terms was at least 1. A false positive gene was called if GO:BP terms were predicted on a gene, but none matched the known, held-out terms (intersection of 0) and false negatives were called on genes that had known, held-out GO:BP terms, but a GO:BP prediction was not made on the gene.

#### Prediction evaluation (semantic similarity)

In addition to precision and recall, we calculated *SS *between the set of held-out terms and predicted terms for the same gene. We employed the *SS *calculation developed by Wang *et al*. [73]. Briefly, each GO term is assigned a semantic value based on the term's location in the GO hierarchy and the relationship types between ancestor GO terms 'is-a' and 'part-of'. The *SS *between two GO terms was calculated by considering the location of both terms in the ontology and the relationships between the ancestor GO terms jointly. *SS *between two sets of GO terms, which is representative of the annotations of two genes, was calculated by iteratively comparing each GO term from the held-out set to the GO terms from the set of predicted terms, and *vice versa*. This method calculates a single *SS *measure on the interval [0,1] for each annotated gene pair compared.

To determine a reliable *SS *threshold, we measured the *SS *between all reported GI gene pairs where each gene in the pair was annotated with at least one GO:BP term. GIs provided the highest *LLS *for any dataset and, therefore, was used as the benchmark set for *SS *scores. The median measure of *SS *for GIs was calculated to be 0.45, which we determined to be the threshold to consider a *SS *score reliable.

#### Prediction evaluation (comparison with sequence similarity)

The translated protein sequences for each of the 483 genes tested were downloaded from FlyBase FB2007_02 [46]. The sequences were searched against the NCBI nr database using BLASTp with an *E*-value cutoff of 10^-6^. Sequence hits with less than 40% identity were removed. Also, all sequences from the *Drosophila *genus were removed. The top 10 BLAST hits for each of the 483 genes were taken and the GO:BP annotations for these BLAST hits were downloaded from the GO database [56]. The mapping between BLAST results and GO term annotations was done through UniProt IDs. All GO:BP annotations were directly transferred to the *D. melanogaster *gene from the top ten BLAST hits.

### Analysis of Teleman *et al*. gene expression data

Processed gene expression data from Teleman *et al*. [79] were downloaded from ArrayExpress [114] under accession number [ArrayExpress:E-TABM-375]. Normalization and filtering was done following the methods in Teleman *et al*. Expression ratios for replicate spots were averaged.

#### Subnetwork construction algorithm

The goal of the subnetwork construction algorithm is to build a tightly connected subnetwork around a set of query genes. This was done by first defining a set of query genes, *Q*. This set is user defined and in this case is a set of genes that share a common biological process. We are given a graph *G *= ⟨*V*, *E*⟩, where *v*_*i*_∈ *V *and *v*_*i*_, *v*_*j*_∈ *E*. In this analysis, *G *=  and *Q *⊂ *V*. We want to find a new set of genes, *Q*', that contains all *v*_*i *_that meet the following criteria: *v*_*i *_∈ *V*, *v*_*i *_∉ *Q*, *v*_*i*_, *v*_*j *_∈ *E*, *v*_*i*_, *v*_*k *_∈ *E*, *v*_*j *_∈ *Q*, and *v*_*k *_∈ *Q*. In other words, we want to find all nodes in *G *that are not already present in *Q *and have an edge between at least two nodes in *Q*. This new set of nodes, *Q*', is then added to *Q *(*Q *= *Q *∪ *Q*'). A second iteration of this procedure is performed to find a new set *Q*' in relation to *Q*. The two sets are again combined to form the final set *Q*. The subnetwork *G*' is returned, where *G*' ⊂ *G *and *G*' = ⟨*Q*, *E*'⟩, *E*' ⊂ *E*.

#### Gene set enrichment analysis

All genes from the wild-type muscle tissue gene expression experiment (fed versus starved larvae) were rank ordered according to their log-transformed ratio values. Gene sets were defined for the following categories: category 1, the functional categories reported in Teleman *et al*.; category 2, the genes from the subnetworks constructed from query seed sets from category 1; category 3, genes listed in KEGG pathways; and category 4, the three GO categories of biological process, molecular function, and cellular component. Gene sets from category 1 were taken directly from the list of genes reported in the figures of Teleman *et al*. Gene sets from category 2 were defined as the genes present in a seed set (gene set in category 1) in addition to the genes from the network constructed according to the subnetwork construction algorithm. Genes that were present in sets from category 1 but not found in the integrated network were not included in any sets in category 2. Gene sets from category 3 were defined by the genes in individual KEGG pathways. Gene sets from category 4 were defined by the genes annotated to individual GO terms. Gene-GO term sets were parsed directly from all associations defined by FlyBase (including IEA, NR, and ND) [56].

The GSEA [81] software was run using the 'GseaPreranked' option, with the rank ordered list of wild-type muscle expression ratios and all gene sets as input. Gene sets smaller than 15 and bigger than 500 were ignored and default weighting parameters were used.

## Abbreviations

AUC: area under the curve; DE: differentially expressed; GEO: Gene Expression Omnibus; GI: genetic interaction; GSEA: gene set enrichment analysis; GO: Gene Ontology; GO:BP: Gene Ontology biological process; *IC*: information content; KEGG: Kyoto Encyclopedia of Genes and Genomes; *LLS*: log-likelihood score; MA: microarray; MRF: Markov random field; ND: No biological data available; NR: not recorded; PPI: protein-protein interaction; SRP: signal recognition particle; *SS*: semantic similarity; *WS*: weighted sum; Y2H: yeast-two-hybrid.

## Authors' contributions

JA conceived the project. MMD, BDE, JCC, and JA were involved in developing the project. JCC, SMB, JRG, RP, and SM performed data processing and computation. JCC, JA, and MMD wrote the paper.

## Additional data files

The following additional data files are available with the online version of this paper: the matrix of values used to create Figure 2 (Additional data file 1); the full set of GO:BP predictions made for  using the MRF method (Additional data file 2); the full set of GO:BP predictions made for  using the MRF method (Additional data file 3); the filtered set of GO:BP predictions made for the 483 genes discussed in the text from  using the MRF method (Additional data file 4); a table of information related to the 70 genes found in Figure 10b (Additional data file 5).

## Supplementary Material

Additional data file 1 The matrix contains GO:BP terms by dataset with the *P*-value reported in each cell. Click here for file

Additional data file 2 All predictions meet the criteria of *t*_*p *_≥ 0.1. Click here for file

Additional data file 3 All predictions meet the criteria of *t*_*p *_≥ 0.1. Click here for file

Additional data file 4 In addition to gene-GO:BP predictions, the file also contains all GO annotations from v5.3 and v5.7 of the *D. melanogaster *genome, best blast hits to the NCBI nr database, and any GO annotations transferred to the gene based on sequence similarity. Genes having any evidence supporting the GO:BP prediction are marked. Click here for file

Additional data file 5A set of signal recognition particle (SRP)-related genes were taken from Teleman *et al*. [79] and a network was built around this query set of genes from the connections in . This file contains information related to gene expression, GO terms, and notes on whether a gene is related to the protein secretory pathway. Networks  and  along with the full set of functional relationships between *Drosophila *gene pairs are made available at [48].Click here for file

## References

[B1] The Gene Ontology Consortium (2008). The Gene Ontology project in 2008.. Nucleic Acids Res.

[B2] Pena-Castillo L, Hughes TR (2007). Why are there still over 1000 uncharacterized yeast genes?. Genetics.

[B3] Watson J, Laskowski R, Thornton J (2005). Predicting protein function from sequence and structural data.. Curr Opin Struct Biol.

[B4] Rost B, Liu J, Nair R, Wrzeszczynski K, Ofran Y (2003). Automatic prediction of protein function.. Cell Mol Life Sci.

[B5] Troyanskaya OG, Dolinski K, Owen AB, Altman RB, Botstein D (2003). A Bayesian framework for combining heterogeneous data sources for gene function prediction (in *Saccharomyces cerevisiae*).. Proc Natl Acad Sci USA.

[B6] Marcotte E, Pellegrini M, Thompson M, Yeates T, Eisenberg D (1999). A combined algorithm for genome-wide prediction of protein function.. Nature.

[B7] Nariai N, Kolaczyk ED, Kasif S (2007). Probabilistic protein function prediction from heterogeneous genome-wide data.. PLoS ONE.

[B8] Jansen R, Yu H, Greenbaum D, Kluger Y, Krogan NJ, Chung S, Emili A, Snyder M, Greenblatt JF, Gerstein M (2003). A Bayesian networks approach for predicting protein-protein interactions from genomic data.. Science.

[B9] Zhu X, Gerstein M, Snyder M (2007). Getting connected: analysis and principles of biological networks.. Genes Dev.

[B10] Fraser AG, Marcotte EM (2004). A probabalistic view of gene function.. Nat Genet.

[B11] Joyce A, Palsson B (2006). The model organism as a system: integrating 'omics' datasets.. Nat Rev Mol Cell Biol.

[B12] Lee I, Date SV, Adai AT, Marcotte EM (2004). A probabilistic functional network of yeast genes.. Science.

[B13] Covert M, Knight E, Reed J, Herrgard M, Palsson B (2004). Integrating high-throughput and computational data elucidates bacterial networks.. Nature.

[B14] DeKeersmaecker S, Thijs I, Vanderleyden J, Marchal K (2006). Integration of omics data: how well does it work for bacteria?. Mol Microbiol.

[B15] Wong SL, Zhang LV, Tong AHY, Li Z, Goldberg DS, King OD, Lesage G, Vidal M, Andrews B, Bussey H, Boone C, Roth FP (2004). Combining biological networks to predict genetic interactions.. Proc Natl Acad Sci USA.

[B16] Lee I, Lehner B, Crombie C, Wong W, Fraser A, Marcotte E (2008). A single gene network accurately predicts phenotypic effects of gene perturbation in *Caenorhabditis elegans*.. Nat Genet.

[B17] Aerts S, Lambrechts D, Maity S, Van Loo P, Coessens B, De Smet F, Tranchevent LC, De Moor B, Marynen P, Hassan B, Carmeliet P, Moreau Y (2006). Gene prioritization through genomic data fusion.. Nat Biotechnol.

[B18] Rhodes D, Yu J, Shanker K, Deshpande N, Varambally R, Ghosh D, Barrette T, Pandey A, Chinnaiyan A (2004). ONCOMINE: a cancer microarray database and integrated data-mining platform.. Neoplasia.

[B19] Franke L, van Bakel H, Fokkens L, de Jong E, Egmont-Petersen M, Wijmenga C (2006). Reconstruction of a functional human gene network, with an application for prioritizing positional candidate genes.. Am J Hum Genet.

[B20] Pujana MA, Han JD, Starita LM, Stevens KN, Tewari M, Ahn JS, Rennert G, Moreno V, Kirchhoff T, Gold B, Assmann V, Elshamy WM, Rual JF, Levine D, Rozek LS, Gelman RS, Gunsalus KC, Greenberg RA, Sobhian B, Bertin N, Venkatesan K, Ayivi-Guedehoussou N, Sole X, Hernandez P, Lazaro C, Nathanson KL, Weber BL, Cusick ME, Hill DE, Offit K (2007). Network modeling links breast cancer susceptibility and centrosome dysfunction.. Nat Genet.

[B21] Aerts S, Vilain S, Hu S, Tranchevent LC, Barriot R, Yan J, Moreau Y, Hassan BA, Quan XJ (2009). Integrating computational biology and forward genetics in *Drosophila*.. PLoS Genet.

[B22] Deng M, Zhang K, Mehta S, Chen T, Shun F (2003). Prediction of protein function using protein-protein interaction data.. J Comput Biol.

[B23] Joshi T, Chen Y, Becker J, Alexandrov N, Xu D (2004). Genome-scale gene function prediction using multiple sources of high-throughput data in yeast *Saccharomyces cerevisiae*.. OMICS.

[B24] Pena-Castillo L, Tasan M, Myers C, Lee H, Joshi T, Zhang C, Guan Y, Leone M, Pagnani A, Kim W, Krumpelman C, Tian W, Obozinski G, Qi Y, Mostafavi S, Lin G, Berriz G, Gibbons F, Lanckriet G, Qiu J, Grant C, Barutcuoglu Z, Hill D, Warde-Farley D, Grouios C, Ray D, Blake J, Deng M, Jordan M, Noble W (2008). A critical assessment of *Mus musculus *gene function prediction using integrated genomic evidence.. Genome Biol.

[B25] Huynen M, Snel B, vanNoort V (2004). Comparative genomics for reliable protein-function prediction from genomic data.. Trends Genet.

[B26] Myers CL, Robson D, Wible A, Hibbs MA, Chiriac C, Theesfeld CL, Dolinski K, Troyanskaya OG (2005). Discovery of biological networks from diverse functional genomic data.. Genome Biol.

[B27] Huttenhower C, Haley EM, Hibbs MA, Dumeaux V, Barrett DR, C oller HA, Troyanskaya OG (2009). Exploring the human genome with functional maps.. Genome Res.

[B28] Lee I, Li Z, Marcotte EM (2007). An improved, bias-reduced probabilistic functional gene network of Baker's yeast, *Saccharomyces cerevisiae*.. PLoS ONE.

[B29] Kemmeren P, Kockelkorn T, Bijma T, Donders R, Holstege F (2005). Predicting gene function through systematic analysis and quality assessment of high-throughput data.. Bioinformatics.

[B30] Gunsalus KC, Ge H, Schetter AJ, Goldberg DS, Han JD, Hao T, Berriz GF, Bertin N, Huang J, Chuang LS, Li N, Mani R, Hyman AA, Sonnichsen B, Echeverri CJ, Roth FP, Vidal M, Piano F (2005). Predictive models of molecular machines involved in *Caenorhabditis elegans *early embryogenesis.. Nature.

[B31] Kim W, Krumpelman C, Marcotte E (2008). Inferring mouse gene functions from genomic-scale data using a combined functional network/classification strategy.. Genome Biol.

[B32] Guan Y, Myers C, Lu R, Lemischka I, Bult C, Troyanskaya O (2008). A genomewide functional network for the laboratory mouse.. PLoS Comput Biol.

[B33] Matthews K, Kaufman T, Gelbart W (2005). Research resources for *Drosophila*: The expanding universe.. Nat Rev Genet.

[B34] Costello J, Cash A, Dalkilic M, Andrews J (2008). Data pushing: a fly-centric guide to bioinformatics tools.. Fly (Austin).

[B35] Tweedie S, Ashburner M, Falls K, Leyland P, McQuilton P, Marygold S, Millburn G, Osumi-Sutherland D, Schroeder A, Seal R, Zhang H, Consortium TF (2009). FlyBase: enhancing *Drosophila *Gene Ontology annotations.. Nucleic Acids Res.

[B36] Lyne R, Smith R, Rutherford K, Wakeling M, Varley A, Guillier F, Janssens H, Ji W, Mclaren P, North P, Rana D, Riley T, Sullivan J, Watkins X, Woodbridge M, Lilley K, Russell S, M A, Mizuguchi K, Micklem G (2007). FlyMine: an integrated database for *Drosophila *and Anopheles genomics.. Genome Biol.

[B37] Spellman PT, Rubin GM (2002). Evidence for large domains of similarly expressed genes in the *Drosophila *genome.. J Biol.

[B38] Samsonova AA, Niranjan M, Russell S, Brazma A (2007). Prediction of gene expression in embryonic structures of *Drosophila melanogaster*.. PLoS Comput Biol.

[B39] Rubin GM, Lewis EB (2000). A brief history of *Drosophila *'s contribution to genome research.. Science.

[B40] Bellen H, Levis R, Liao G, He Y, Carlson J, Tsang G, Evans-Holm M, Hiesinger P, Schulze K, Rubin G, Hoskins R, Spradling A (2004). The BDGP gene disruption project: single transposon insertions associated with 40% of *Drosophila *genes.. Genetics.

[B41] Dietzl G, Chen D, Schnorrer F, Su K, Barinova Y, Fellner M, Gasser B, Kinsey K, Oppel S, Scheiblauer S, Couto A, Marra V, Keleman K, Dickson B (2007). A genome-wide transgenic RNAi library for conditional gene inactivation in *Drosophila*.. Nature.

[B42] Stark A, Lin M, Kheradpour P, Pederson J, Parts L, Carlson J, Crosby M, Rasmussen M, Roy S, Deogras A, Ruby J, Brennecke J, Hodges E, Hinrichs A, Caspi A, Paten B, Park S, Han M, Maeder M, Polansky B, Robson B, Aerts S, vanHelden J, Hassan B, Gilbert D, Eastman D, Rice M, Weir M, Harvard FlyBase curators, Berkeley Drosophila Genome Project (2007). Discovery of functional elements in 12 *Drosophila *genomes using evolutionary signatures.. Nature.

[B43] Clark A, Eisen M, Smith D, Bergman C, Oliver B, Markow T, Kaufman T, Kellis M, W G, Iyer V, Pollard D, Sackton T, Larracuente A, Singh N, Abad J, Abt D, Adryan B, Aguade M, Akashi H, Andreson W, Aguadro C, Ardell D, Arguello R, Artieri C, Barbash D, Barker D, Barsanti P, Batterham P, Batzoglou S, Drosophila 12 Genomes Consortium (2007). Evolution of genes and genomes on the *Drosophila *phylogeny.. Nature.

[B44] Celniker S, Dillon L, Gerstein M, Gunsalus K, Henikoff S, Karpen G, Kellis M, Lai E, Lieb J, MacAlpine D, Micklem G, Piano F, Snyder M, Stein KL White, Waterson R, modENCODE Consortium (2009). Unlocking the secrets of the genome.. Nature.

[B45] The ENCODE Project Consortium (2004). The ENCODE(ENCyclopedia Of DNA Elements).. Science.

[B46] FlyBase. http://www.flybase.net.

[B47] Letovsky S, Kasif S (2003). Predicting protein function from protein/protein interaction data: a probabilistic approach.. Bioinformatics.

[B48] Supplemental. http://www.indigene.org.

[B49] Bader GD, Donaldson I, Wolting C, Ouellette BFF, Pawson T, Hogue CWV (2001). BIND - The Biomolecular Interaction Network Database.. Nucleic Acids Res.

[B50] Xenarios I, Salwinski L, Duan XJ, Higney P, Kim SM, Eisenberg D (2002). DIP, the Database of Interacting Proteins: a research tool for studying cellular networks of protein interactions.. Nucleic Acids Res.

[B51] Stanyon CA, Liu G, Mangiola BA, Patel N, Giot L, Kuang B, Zhang H, Zhong J, Finley RL (2004). A *Drosophila *protein-interaction map centered on cell-cycle regulators.. Genome Biol.

[B52] Stark C, Breitkreutz BJ, Reguly T, Boucher L, Breitkreutz A, Tyers M (2006). BioGRID: a general repository for interaction datasets.. Nucleic Acids Res.

[B53] Kerrien S, Alam-Faruque Y, Aranda B, Bancarz I, Bridge A, Derow C, Dimmer E, Feuermann M, Friedrichsen A, Huntley R, Kohler C, Khadake J, Leroy C, Liban A, Lieftink C, Montecchi-Palazzi L, Orchard S, Risse J, Robbe K, Roechert B, Thorneycroft D, Zhang Y, Apweiler R, Hermjakob H (2007). IntAct-open source resource for molecular interaction data.. Nucleic Acids Res.

[B54] Giot L, Bader JS, Brouwer C, Chaudhuri A, Kuang B, Li Y, Hao YL, Ooi CE, Godwin B, Vitols E, Vijayadamodar G, Pochart P, Machineni H, Welsh M, Kong Y, Zerhusen B, Malcolm R, Varrone Z, Collis A, Minto M, Burgess S, McDaniel L, Stimpson E, Spriggs F, Williams J, Neurath K, Ioime N, Agee M, Voss E, Furtak K (2003). A protein interaction map of *Drosophila melanogaster*.. Science.

[B55] Supplemental Figures and Tables. http://www.indigene.org/downloads/Costello_Suppl_Data.pdf.

[B56] Gene Ontology. http://www.geneontology.org/.

[B57] De Gregorio E, Spellman PT, Tzou P, Rubin GM, Lemaitre B (2002). The Toll and Imd pathways are the major regulators of the immune response in *Drosophila*.. EMBO J.

[B58] Wertheim B, Kraaijeveld AR, Schuster E, Blanc E, Hopkins M, Pletcher SD, Strand MR, Partridge L, Godfray HC (2005). Genome-wide gene expression in response to parasitoid attack in *Drosophila*.. Genome Biol.

[B59] Magalhaes TR, Palmer J, Tomancak P, Pollard KS (2007). Transcriptional control in embryonic *Drosophila *midline guidance assessed through a whole genome approach.. BMC Neurosci.

[B60] Deng M, Sun F, Chen T (2003). Assessment of the reliability of protein-protein interactions and protein function prediction.. Pac Symp Biocomput.

[B61] Arbeitman MN, Furlong EE, Imam F, Johnson E, Null BH, Baker BS, Krasnow MA, Scott MP, Davis RW, White KP (2002). Gene expression during the life cycle of *Drosophila melanogaster*.. Science.

[B62] Kanehisa M, Goto S (2000). KEGG: Kyoto Encyclopedia of Genes and Genomes.. Nucleic Acids Res.

[B63] Integrated *Drosophila *Gene Network with 20 K Edges. http://www.indigene.org/downloads/Costello_20K_network.cys.

[B64] Integrated *Drosophila *Gene Network with 200 K Edges. http://www.indigene.org/downloads/Costello_200K_network.cys.

[B65] Full Set of Integrated *Drosophila *Data. http://www.indigene.org/downloads/Costello_All_Data.tar.gz.

[B66] Shannon P, Markiel A, Ozier O, Baliga N, Wang J, Ramage D, Amin N, Schwikowski B, Ideker T (2003). Cytoscape: a software environment for integrated models of biomolecular interaction networks.. Genome Res.

[B67] Bader G, Hogue C (2003). An automated method for finding molecular complexes in large protein interaction networks.. BMC Bioinformatics.

[B68] Guan X, Middlebrooks B, Alexander S, Wasserman S (2006). Mutation of TweedleD, a member of an unconventional cuticle protein family, alters body shape in *Drosophila*.. Proc Natl Acad Sci USA.

[B69] Kadrmas J, Smith M, Pronovost S, Beckerle M (2007). Characterization of RACK1 function in *Drosophila *development.. Dev Dyn.

[B70] Shor B, Calaycay J, Rushbrook J, McLeod M (2003). Cpc2/RACK1 is a ribosome-associated protein that promotes efficient translation in *Schizosaccharomyces pombe*.. J Biol Chem.

[B71] Gerbasi V, Weaver C, Hill S, Friedman D, Link A (2004). Yeast Asc1p and mammalian RACK1 are functionally orthologous core 40S ribosomal proteins that repress gene expression.. Mol Cell Biol.

[B72] Sengupta J, Nilsson J, Gursky R, Spahn C, Nissen P, Frank J (2004). Identification of the versatile scaffold protein RACK1 on the eukaryotic ribosome by cryo-EM.. Nat Struct Mol Biol.

[B73] Wang JZ, Du Z, Payattakool R, Yu PS, Chen CF (2007). A new method to measure the semantic similarity of GO terms.. Bioinformatics.

[B74] Schuh M, Lehner S, Heidmann S (2007). Incorporation of *Drosophila *CID/CENP-A and CENP-C into centromeres during early embryonic anaphase.. Curr Biol.

[B75] Bridges C (1920). The mutant crossveinless in *Drosophila melanogaster*.. Proc Natl Acad Sci USA.

[B76] Shimmi O, Ralston A, Blair S, O'Connor M (2005). The *crossveinless *gene encodes a new member of the Twisted gastrulation family of BMP-binding proteins which, with Short gastrulation, promotes BMP signaling in the crossveins of the *Drosophila *wing.. Dev Biol.

[B77] Allison D, Cui X, Page G, Sabripour M (2006). Microarray data analysis: from disarray to consolidation and consensus.. Nat Rev Genet.

[B78] Huang D, Sherman B, Lempicki R (2009). Bioinformatics enrichment tools: paths toward the comprehensive functional analysis of large gene lists.. Nucleic Acids Res.

[B79] Teleman A, Hietakangas V, Sayadian A, Cohen S (2008). Nutritional control of protein biosynthetic capacity by insulin via Myc in *Drosophila*.. Cell Metab.

[B80] Abrams EW, Andrew DJ (2005). CrebA regulates secretory activity in the *Drosophila *salivary gland and epidermis.. Development.

[B81] Subramanian A, Tamayo P, Mootha V, Mukherjee S, Ebert B, Gillette M, Paulovich A, Pomeroy SL, Golub T, Lander E, Mesirova J (2005). Gene set enrichment analysis: A knowledge-based approach for interpreting genome-wide expression profiles.. Proc Natl Acad Sci USA.

[B82] Ioannidis JP, Allison DB, Ball CA, Coulibaly I, Cui X, Culhane AC, Falchi M, Furlanello C, Game L, Jurman G, Mangion J, Mehta T, Nitzberg M, Page GP, Petretto E, van Noor V (2009). Repeatability of published microarray gene expression analyses.. Nat Genet.

[B83] Sprinzak E, Sattath S, Margalit H (2003). How reliable are experimental protein-protein interaction data?. J Mol Biol.

[B84] Lècuyer E, Yoshida H, Parthasarathy N, Alm C, Babak T, Cerovina T, Hughes T, Tomancak P, Krause H (2007). Global analysis of mRNA localization reveals a prominent role in organizing cellular architecture and function.. Cell.

[B85] Halfon M, Gallo S, Bergman C (2008). REDfly 2.0: an integrated database of cis-regulatory modules and transcription factor binding sites in *Drosophila*.. Nucleic Acids Res.

[B86] Bergman CM, Carlson JW, Celniker SE (2005). *Drosophila *DNase I footprint database: a systematic genome annotation of transcription factor binding sites in the fruitfly, *Drosophila melanogaster*.. Bioinformatics.

[B87] Flockhart I, Booker M, Kiger A, Boutros M, Armknecht S, Ramadan N, Richardson K, Xu A, Perrimon N, Mathey-Prevot B (2006). FlyRNAi: the *Drosophila *RNAi screening center database.. Nucleic Acids Res.

[B88] Huynen M, Snel B, Lathe W, Bork P (2000). Predicting protein function by genomic context: quantitative evaluation and qualitative inferences.. Genome Res.

[B89] Pellegrini M, Marcotte E, Thompson M, Eisenberg D, Yeates T (1999). Assigning protein functions by comparative genome analysis: protein phylogenetic profiles.. Proc Natl Acad Sci USA.

[B90] Bowers P, Pellegrini M, Thompson M, Fierro J, Yeates T, Eisenberg D (2004). Prolinks: a database of protein functional linkages derived from coevolution.. Genome Biol.

[B91] Li TR, White KP (2003). Tissue-specific gene expression and ecdysone-regulated genomic networks in *Drosophila*.. Dev Cell.

[B92] Parisi M, Nuttall R, Naiman D, Bouffard G, Malley J, Andrews J, Eastman S, Oliver B (2003). Paucity of genes on the *Drosophila *X chromosome showing male-biased expression.. Science.

[B93] Estrada B, Choe SE, Gisselbrecht SS, Michaud S, Raj L, Busser BW, Halfon MS, Church GM, Michelson AM (2006). An integrated strategy for analyzing the unique developmental programs of different myoblast subtypes.. PLoS Genet.

[B94] Qin X, Ahn S, Speed TP, Rubin GM (2007). Global analyses of mRNA translational control during early *Drosophila *embryogenesis.. Genome Biol.

[B95] Beckstead RB, Lam G, Thummel CS (2005). The genomic response to 20-hydroxyecdysone at the onset of *Drosophila *metamorphosis.. Genome Biol.

[B96] Sorensen JG, Nielsen MM, Kruhoffer M, Justesen J, Loeschcke V (2005). Full genome gene expression analysis of the heat stress response in *Drosophila melanogaster*.. Cell Stress Chaperones.

[B97] Hild M, Beckmann B, Haas SA, Koch B, Solovyev V, Busold C, Fellenberg K, Boutros M, Vingron M, Sauer F, Hoheisel JD, Paro R (2003). An integrated gene annotation and transcriptional profiling approach towards the full gene content of the *Drosophila *genome.. Genome Biol.

[B98] Altenhein B, Becker A, Busold C, Beckmann B, Hoheisel JD, Technau GM (2006). Expression profiling of glial genes during *Drosophila *embryogenesis.. Dev Biol.

[B99] Edwards AC, Rollmann SM, Morgan TJ, Mackay TF (2006). Quantitative genomics of aggressive behavior in *Drosophila melanogaster*.. PLoS Genet.

[B100] Hooper SD, Boué S, Krause R, Jensen LJ, Mason CE, Ghanim M, White KP, Furlong EE, Bork P (2007). Identification of tightly regulated groups of genes during *Drosophila melanogaster *embryogenesis.. Mol Syst Biol.

[B101] Sandmann T, Jensen LJ, Jakobsen JS, Karzynski MM, Eichenlaub MP, Bork P, Furlong EE (2006). A temporal map of transcription factor activity: *mef2 *directly regulates target genes at all stages of muscle development.. Dev Cell.

[B102] Chintapalli VR, Wang J, Dow JA (2007). Using FlyAtlas to identify better *Drosophila melanogaster *models of human disease.. Nat Genet.

[B103] Tomancak P, Beaton A, Weiszmann R, Kwan E, Shu S, Lewis SE, Richards S, Ashburner M, Hartenstein V, Celniker SE, Rubin GM (2002). Systematic determination of patterns of gene expression during *Drosophila *embryogenesis.. Genome Biol.

[B104] The R Project for Statistical Computing. http://www.r-project.org/.

[B105] Futschik ME, Crompton T (2005). OLIN: optimized normalization, visualization and quality testing of two-channel microarray data.. Bioinformatics.

[B106] Affymetrix (2001). Affymetrix Microarray Suite User's Guide Version 50.

[B107] Wu Z, Irizarry R, Gentleman R, Martinez-Murillo F, Spencer F (2001). A model-based background adjustment for oligonucleotide expression arrays.. J Am Stat Assoc.

[B108] Affymetrix *Drosophila *Platform Files. http://www.affymetrix.com/support/technical/byproduct.affx?cat=arrays.

[B109] Troyanskaya O, Cantor M, Sherlock G, Brown P, Hastie T, Tibshirani R, Botstein D, Altman R (2001). Missing value estimation methods for DNA microarrays.. Bioinformatics.

[B110] Lord P, Stevens R, Brass A, Goble C (2003). Semantic similarity measures as tools for exploring the Gene Ontology.. Pac Symp Biocomput.

[B111] Eisen MB, Spellman PT, Brown PO, Botstein D (1998). Cluster analysis and display of genome-wide expression patterns.. Proc Natl Acad Sci USA.

[B112] Saeed A, Sharov V, White J, Li J, Liang W, Bhagabati N, Braisted J, Klapa M, Currier T, Thiagarajan M, Sturn A, Snuffin M, Rezantsev A, Popov D, Ryltsov A, Kostukovich E, Borisovsky I, Liu Z, Vinsavich A, Trush V, Quackenbush J (2003). TM4: a free, open-source system for microarray data management and analysis.. Biotechniques.

[B113] KEGG *Drosophila *Download FTP Directory. ftp://ftp.genome.jp/pub/kegg/pathway/organisms/dme/.

[B114] ArrayExpress. http://www.ebi.ac.uk/microarray-as/ae/.

